# Enteropathogenic *Escherichia coli* induces *Entamoeba histolytica* superdiffusion movement on fibronectin by reducing traction forces

**DOI:** 10.1371/journal.ppat.1012618

**Published:** 2025-05-23

**Authors:** Yuanning Guo, Jun Ye, Ariel Shemesh, Anas Odeh, Meirav Trebicz-Geffen, Haguy Wolfenson, Serge Ankri

**Affiliations:** 1 Department of Genetics and Developmental Biology, Rappaport Faculty of Medicine, Technion – Israel Institute of Technology, Haifa, Israel; 2 Department of Molecular Microbiology, Rappaport Faculty of Medicine, Technion – Israel Institute of Technology, Haifa, Israel; 3 Biomedical core facilities, Rappaport Faculty of Medicine, Technion – Israel Institute of Technology, Haifa, Israel; University of Utah, UNITED STATES OF AMERICA

## Abstract

Amebiasis, caused by *Entamoeba histolytica*, is a global health concern, affecting millions and causing significant mortality, particularly in areas with poor sanitation. Although recent studies have examined *E. histolytica*’s interaction with human intestinal microbes, the impact of bacterial presence on the parasite’s motility, mechanical forces, and their potential role in altering invasiveness have not been fully elucidated. In this study, we utilized a micropillar-array system combined with live imaging to investigate the effects of enteropathogenic *Escherichia coli* on *E. histolytica*’s motility characteristics, F-actin spatial localization, and traction force exerted on fibronectin-coated substrates. Our findings indicate that co-incubation with live enteropathogenic *E. coli* significantly enhances the motility of *E. histolytica*, as evidenced by superdiffusive movement—characterized by increased directionality and speed—resulting in broader dispersal and more extensive tissue/cell damage. This increased motility is accompanied by a reduction in F-actin-dependent traction forces and podosome-like structures on fibronectin-coated substrates, but with increased F-actin localization in the upper part of the cytoplasm. These findings highlight the role of physical interactions and cellular behaviors in modulating the parasite’s virulence, providing new insights into the mechanistic basis of its pathogenicity.

## Author summary

Amebiasis, caused by the protozoan parasite *Entamoeba histolytica*, is a major global health issue, affecting around 50 million people and resulting in 100,000 deaths annually. The disease is transmitted through contaminated food and water. Upon reaching the intestines, which are teeming with bacteria, *E. histolytica* begins its invasion by removing the protective mucus layer, followed by adhering to and detaching enterocytes, leading to the disruption and degradation of the epithelial barrier. Afterwards, *E. histolytica* invades along the fibronectin-rich basement membrane deep into the crypts of Lieberkühn, eventually penetrating the fibronectin-rich lamina propria and even deeper layers. This leads to further tissue destruction and potential dissemination to distant organs, causing severe complications. In our study, we explored how the presence of enteropathogenic bacteria affects the parasite’s motility and mechanical force generation, both of which are key to its pathogenicity. Using a micropillar-array system and live imaging, we found that exposure to live enteropathogenic *Escherichia coli* significantly increases *E. histolytica*’s motility while reducing its traction force on fibronectin-rich matrices. These changes in behavior highlight the role of bacterial interactions in enhancing the parasite’s virulence. Our findings provide important insights into the mechanistic basis of *E. histolytica*’s pathogenicity, offering potential avenues for new treatments against amebiasis.

## Introduction

Amebiasis, caused by the protozoan parasite *Entamoeba histolytica*, is a parasitic infection that affects approximately 50 million people and causes 100,000 deaths each year worldwide, especially in regions with inadequate sanitation and hygiene practices [1,2]. The transmission of this parasitic infection occurs via the fecal-oral route, predominantly through contaminated food or water containing *E. histolytica* cysts (the infective form). Upon entry into the host’s intestine, the cyst undergoes excystation, releasing trophozoites (the invasive form) that can invade the colonic mucosa, leading to tissue destruction and abscess formation [3,4].

Invasive intestinal disease caused by *E. histolytica* can manifest as abdominal pain and bloody diarrhea, corresponding histologically with trophozoites invading and undermining the intestinal surface laterally, forming the characteristic flask-shaped ulcers. Rarely, *E. histolytica* trophozoites can enter the bloodstream and disseminate to other organs, most often to the liver, where they result in tissue destruction with inflammation, forming amebic liver abscesses [4,5].

The invasiveness and virulence are largely dependent on motility and physical forces exerted by *E. histolytica*, particularly through mechanisms of adherence, tissue invasion, and cytotoxic activities. The motility of *E. histolytica* is powered by its dynamic actomyosin cytoskeleton, which includes fibrous actin (F-actin) and myosin proteins [6,7]. The parasite’s movement, particularly characterized by amoeboid migration, is facilitated not only by the formation of pseudopods at the leading edge (driven by local actin assembly), but also by contraction at the rear, generating pressure propelled by actin and myosin II. These motility properties enable the parasite to navigate through host microenvironment and tissues, i.e., intestinal mucus and epithelia, extracellular matrix (ECM), and blood vessels [8,9]. Following the degradation of the mucus layer and the removal of epithelial cells, *E. histolytica* migrates across the fibronectin-rich basement membrane towards the crypts of Lieberkühn, before invading the lamina propria and deeper structures [10]. During this migration and invasion, *E. histolytica* trophozoites adhere to the ECM and form adhesions to fibronectin by interacting with it via a β1-integrin-like receptor [11–13]. Traction force is generated by the actin cytoskeleton and transmitted to the ECM through these adhesions [14–16].

The human intestine harbors an estimated 10^13–10^14 microbes across over 1,000 identified microbial species [17–19]. The interactions among these microbes, as well as between the microbes and the host, are highly complex and frequent, playing a crucial role in intestinal physiology and pathology [19]. Notably, the crosstalks between *E. histolytica*, the host digestive and immune systems, as well as the microbiota that influence amebiasis are emerging as a significant area of research [20,21]. For instance, multiple interactions between *E. histolytica* and bacterial biofilms may affect disease persistence and antibiotic resistance [21]. The composition of the gut microbiota varies among individuals with different clinical manifestations of *E. histolytica* infection, such as asymptomatic colonization, colitis, and liver abscess [22–24]. Normal microbiota can protect the host from amebic colitis by recruiting neutrophils to the gut via the CXCR2 pathway [24,25]. Furthermore, the metabolite deoxycholic acid produced by *Clostridium scindens* has been shown to increase granulocyte-monocyte progenitors in the bone marrow, which in turn elevates colonic neutrophil levels during amebic infection [20,26]. Additionally, segmented filamentous bacteria, another commensal, interact with bone marrow dendritic cells through serum amyloid A, leading to the recruitment of colonic neutrophils, dendritic cells, and upregulation of interleukin-17A as part of the immune defense against amebiasis [20,27].

Still, research on the direct interactions between *E. histolytica* and other microbes is limited, highlighting the urgent need for further investigation. These interactions, accompanied by *E. histolytica* invading and destroying the intestinal mucosa layers, significantly influence the parasite’s invasiveness and virulence [19]. Several in vitro studies have demonstrated that the presence of enteropathogenic bacteria enhances the pathogenic behavior of *E. histolytica*. For instance, interaction with virulent bacteria such as enteropathogenic *Escherichia coli*, *Shigella dysenteriae*, or *Clostridium symbiosum* can increase the parasite’s cysteine proteases activity, cytopathic effect, erythrophagocytosis, hemolytic activity, and host’s proinflammatory cytokines secretion [28–31]. However, whether the interaction between bacteria and *E. histolytica* has a crucial impact on the motility and physical force of the parasites, and thus further affects *E. histolytica*’s invasiveness, remains unknown.

In this study, we employed a micropillar-array system [32] combined with live imaging to investigate whether and how enteropathogenic *E. coli* affect the traction forces exerted by *E. histolytica* on fibronectin substrates. By analyzing changes in traction forces, we aimed to uncover how these alterations affect the motility and invasiveness of the parasite. Our investigation seeks to illuminate the role of physical interactions and cellular behaviors in the virulence of *E. histolytica*, offering new insights into the mechanistic basis of its pathogenicity.

## Results

### *E. histolytica* generates significantly higher traction force on fibronectin-coated micropillars compared to uncoated ones.

**Fig 1:**
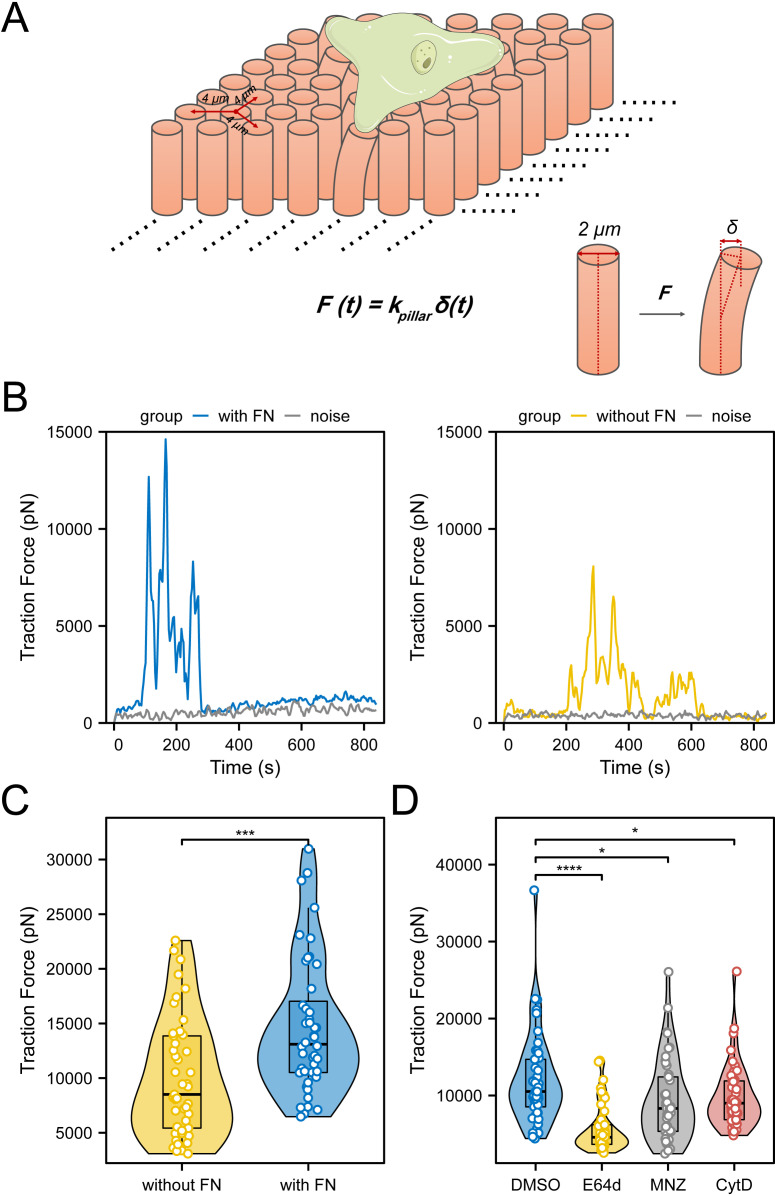
*E. histolytica* generates significantly larger traction forces on fibronectin-coated micropillars compared to uncoated ones. (A) Schematic diagram showing how a trophozoite interacts with the micropillar-array surface and thereby generating traction force, leading to the deflection of micropillars. The traction force as a function of *F(t)* is calculated by multiplying the spring constant of a micropillar *k*_*pillar*_ and micropillar displacement over time *δ(t)*. The cartoon illustration of the *E. histolytica* trophozoite was adopted and modified from Servier Medical Art (smart.servier.com), licensed under CC BY 4.0. (B) Representative graphs showing the temporal changing of traction force on a micropillar as a trophozoite passing on a micropillar with (left) or without (right) fibronectin (FN) coating. The grey curve indicates background noise of micropillars untouched by the cells. (C) The maximum traction force exerted by *E. histolytica* on micropillars was compared between fibronectin-coated (n = 44 from 9 cells) and uncoated micropillars (n = 45 from 9 cells) using t-test. (D) The maximum traction force exerted by *E. histolytica* trophozoites on fibronectin-coated micropillars was analyzed between DMSO-treated controls and those treated with the cysteine protease inhibitor E64d, metronidazole (MNZ), or the F-actin polymerization inhibitor Cytochalasin D (CytD), using the Kruskal-Wallis test followed by post-hoc pairwise comparisons. The analyses included 45 micropillars from 9 cells for each treatment condition, with 5 pillars analyzed per cell. * *P *< 0.05, *** *P* < 0.001, **** *P* < 0.0001.

Polydimethylsiloxane (PDMS) micropillar-array is an effective system to study cellular traction forces across a range of physiological stiffnesses, including those found in gut tissues encountered by *E. histolytica* trophozoites due to the excellent biocompatibility and high structural flexibility of PDMS [32–34]. The elastic PDMS micropillars provide a surface for cells to adhere to and can be bent by the cells. Tracking cell movements and pillar displacements with live-cell imaging enables the analysis of cell motility and cellular forces (Fig 1A, see Materials and methods section for details). We coated the surface micropillar-arrays with human fibronectin, as interaction with the ECM, particularly fibronectin, plays a paramount role in *E. histolytica* invasion and virulence [35,36]. Indeed, histology slides from the Human Protein Atlas (HPA, www.proteinatlas.org) [37] revealed that fibronectin is abundant in human intestine, with particularly prominent expression in the subepithelial basement membrane and lamina propria of mucosa (S1 Fig).

We have tested several arrays of micropillars with the same diameter, 2 μm, and different heights including 5.3, 9.4, and 13.2 μm, corresponding to rigidities (spring constant, *k*_*pillar*_) of 31, 6 and 2 pN/nm, respectively, calculated by Euler-Bernoulli beam theory [38]. Consequently, only the micropillar-array of height 5.3 μm was employed in this study, since *E. histolytica* generated forces that led to very large micropillars deflections on softer/higher micropillars-arrays (9.4 and 13.2 μm height), resulting in tracking difficulties for later analyses. The effective elastic modulus of the micropillars-array of height 5.3 μm is approximately 22.7 kPa, which is within the human physiological range [39].

To assess the biomechanical relevance of this system, we first compared the traction forces applied by *E. histolytica* trophozoites on pillars with and without fibronectin coating. Curves of traction force as a function of time *F(t)* of specific micropillars were plotted showing the magnitude and characteristics of cellular force (Fig 1B). For comparisons between different conditions, we used the maximum force value from each curve, representing the highest traction force exerted by the cell on the corresponding micropillar. The results show that *E. histolytica* trophozoites apply significantly greater traction forces on fibronectin-coated micropillars compared to uncoated ones. (Fig 1B and 1C).

Analyses of the motility properties of *E. histolytica* on the micropillars—including measurements of the Directionality Ratio, Average Speed, Maximum Euclidean Displacement (MED) Speed, and Normalized Area Coverage Rate (see definitions of these quantities and details on cell tracking and segmentation for live-imaging videos in the Materials and methods section)—revealed higher motility on fibronectin-coated compared to uncoated micropillars (S2 Fig).

Next, we employed three inhibitors and/or medications for *E. histolytica* that are expected to affect cellular force generation on fibronectin-coated micropillars by influencing cell adhesion and the actin cytoskeleton. The treatment concentrations for *E. histolytica* were determined based on published literature [40–42]. Only visibly active migrating cells observed in the live-imaging videos were included in the analysis. We applied the F-actin polymerization inhibitor Cytochalasin D (CytD) since traction force generation depends on F-actin cytoskeleton contraction and adhesion formation on ECM [14–16]. We used the cysteine protease inhibitor E64d since E64 derivatives can significantly decrease the adhesion of *E. histolytica* to ECM proteins [43]. We also applied the antibiotic metronidazole (MNZ), a drug currently used to treat amebiasis, as its treatment induces the formation of oxidized proteins in *E. histolytica*, including cytoskeletal proteins such as actin. This oxidative stress not only impairs the formation of F-actin [42], but also reduces the adhesion of *E. histolytica* to substrate [44].

Traction forces exerted by *E. histolytica* on fibronectin-coated micropillars significantly decreased when treated with these inhibitors (Fig 1D). Taken together, depriving the ECM component fibronectin from the substrate, preventing F-actin polymerization (CytD), suppressing cysteine protease (E64d), and inducing oxidative stress (MNZ), all significantly reduce traction forces of *E. histolytica* on the external substrate.

### Exposure to enteropathogenic *E. coli* led to significant alterations in both motility properties and cellular contraction force of *E. histolytica.*

**Fig 2:**
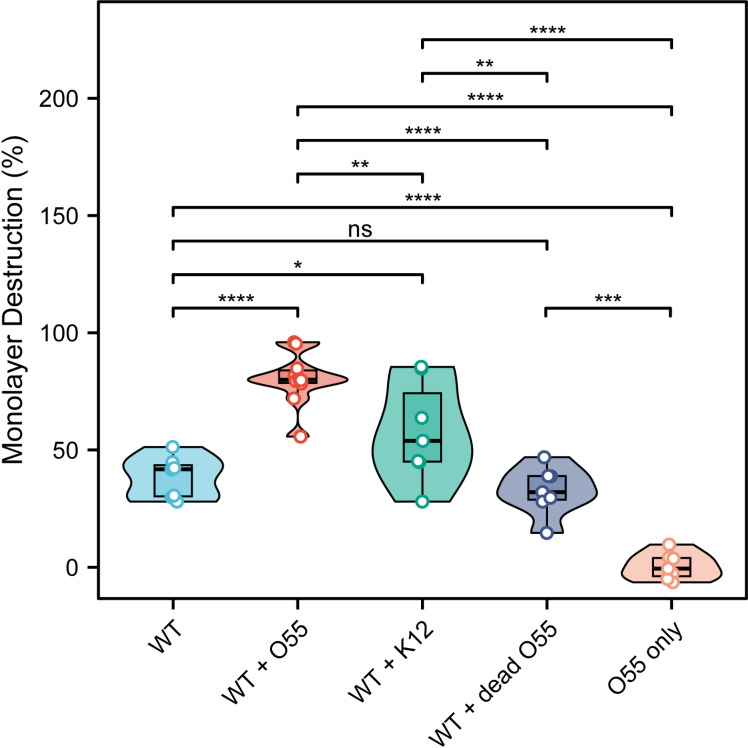
Cytopathic assay of *E. histolytica* trophozoites in different conditions following short period of *E. coli* exposure. The cytopathic behavior of *E. histolytica* trophozoites and *E. coli* in different combinations, including WT (wild type, no bacterial exposure) trophozoites (n = 7), enteropathogenic *E. coli* O55 only (n = 10), as well as trophozoites incubated with enteropathogenic *E. coli* O55 (n = 10), dead *E. coli* O55 (n = 7), and nonpathogenic *E. coli* K12 (n = 7), was assessed and compared based on their ability to destroy a monolayer of HeLa cells. Statistical analyses, one-way ANOVA with post-hoc pairwise comparisons. * *p *< 0.05, ** *P* < 0.01, *** *P* < 0.001, **** *P* < 0.0001.

*E. histolytica* resides in a bacteria-rich environment within the intestine, where it not only interacts with but also feeds on various bacterial species, which in turn can modulate its virulence [45,46]. We performed cytopathic assays to compare the ability of *E. histolytica* trophozoites to destroy a HeLa cell monolayer across different groups after a short co-incubation period (30 minutes) with various *E. coli* strains, including enteropathogenic O55 and nonpathogenic K12. Control conditions included WT (wild type, no bacterial exposure), WT co-incubated with paraformaldehyde (PFA)-fixed (dead) O55, as well as seeding O55 alone onto the monolayer without trophozoites. The results confirmed that co-incubation of trophozoites with live *E. coli* resulted in a greater percentage of destruction of a HeLa cell monolayer, with the enteropathogenic O55 strain showing the highest ability in enhancing the parasite’s destructive effect (Fig 2).

To better replicate the conditions *E. histolytica* trophozoites encounter during the invasion along fibronectin-rich basement membrane beneath the human intestine epithelium, as well as the further invasion to the lamina propria and even deeper structures (S1 Fig), our subsequent experiments were all conducted using human fibronectin-coated micropillars.

To investigate the impact of bacterial exposure on the invasiveness of *E. histolytica*, which is partly manifested through changes in motility, we pre-incubated the trophozoites with *E. coli* (enteropathogenic O55 or nonpathogenic K12) at 37°C for 30 minutes, using the same conditions as the previously described cytopathic assay. This was followed by live imaging using the micropillar system. We then analyzed and compared the motility features of trophozoites under three conditions: 1) WT control (no bacterial exposure), 2) incubation with *E. coli* O55, and 3) incubation with *E. coli* K12. Additional control experiments included trophozoite incubation with dead *E. coli* O55, as well as with fibroblasts, which are abundant and widely distributed stromal cells throughout the intestinal layers, making them highly likely to interact with trophozoites.

To obtain accurate positional, area, and morphological information of cells, we developed and employed a two-phase artificial intelligence (AI)-based tracking and segmentation workflow to track and segment cells in live-imaging videos. This method effectively ensures accuracy and avoids interference from complex backgrounds.

**Fig 3:**
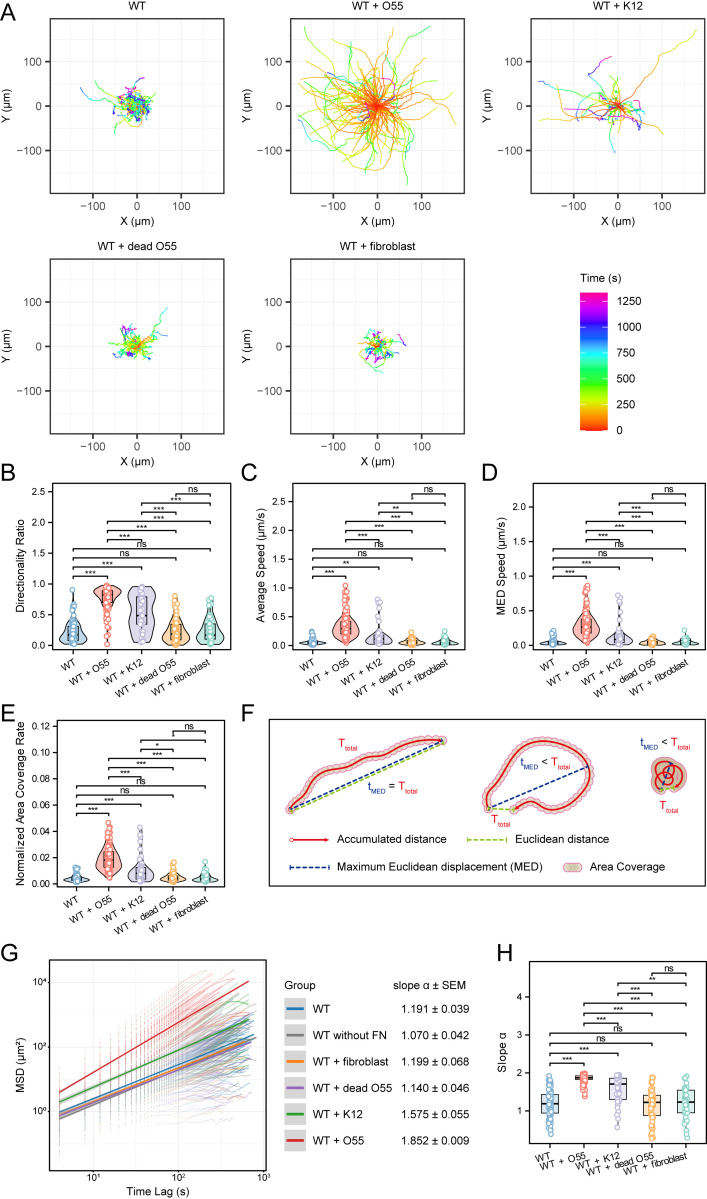
Significant differences in motility properties of *E. histolytica* following short period of *E. coli* exposure. (A) Trajectory plots of trophozoite migration under different conditions: WT control (n = 82), incubation with enteropathogenic *E. coli* O55 (n = 141), incubation with nonpathogenic *E. coli* K12 (n = 41), incubation with dead *E. coli* O55 (n = 79), as well as incubation with fibroblasts (n = 37). Note that WT + O55 group spreads and occupies the entire plot within a relatively short time (red and yellow), whereas the other groups, even over a prolonged period (cyan, blue, and purple), remain confined to a limited area, clearly indicating that the WT + O55 group reaches a greater distance in less time. (B-E) Quantitative comparison of the migration Directionality Ratio (B), Average Speed (C), Maximum Euclidean Displacement (MED) Speed (D), and Normalized Area Coverage Rate (by time and cell size) (E) of *E. histolytica* under the different conditions described in (A), with Kruskal-Wallis test and post-hoc pairwise comparisons. (F) The schematic diagram illustrates Accumulated Distance, Euclidean Distance, MED, and Area Coverage of cells (with cell overlapping regions not counted multiple times) along with their corresponding time periods. The parameters in Fig B-E are derived from these calculations (details are in Materials and methods section). (G) The relationship between mean square displacements (MSD) and the corresponding time (seconds) was plotted on a logarithmic scale for *E. histolytica* locomotion under the different conditions. The solid lines and shaded areas represent the linear fit for each group and the corresponding 95% confidence interval. The slopes (α) and standard error of the mean (SEM) from the linear fits are summarized on the right. (F) Comparison of the slopes (α) for each individual cell/plot under the different conditions, with Kruskal-Wallis test with post-hoc pairwise comparisons Notably, the average slope α of O55 group is the highest, indicating that it exhibits the strongest superdiffusive movement pattern. * *P* < 0.05, ** *P* < 0.01, *** *P* < 0.001.

Analyses of movement trajectories revealed that trophozoites exposed to bacteria migrated significantly farther than the control groups, with those incubated with enteropathogenic *E. coli* O55 exhibiting the highest speed and covering the greatest distance in the shortest time (Fig 3A and S3 Fig, see also S1 and S2 Videos). The median recorded durations were 1030, 324, 560, 640, 740 seconds for WT control, WT + O55, WT + K12, WT + dead O55, and WT + fibroblasts, respectively. Further quantitative analyses indicated that the migration Average Speed, Maximum Euclidean Displacement Speed (MED speed, defined as Maximum Euclidean Displacement divided by the corresponding time), Directionality Ratio, and Normalized Area Coverage Rate (by time and cell size) of trophozoites incubated with live *E. coli* O55 were all significantly higher than the other groups (Fig 3B–F). The motility characteristics of trophozoites incubated with *E. coli* K12 were intermediate between the control and those incubated with *E. coli* O55. There was no significant change in *E. histolytica* motility when co-incubated with fibroblasts compared to the WT control group.

To better understand the cell migration mode, we calculated the mean square displacements (MSD) of the cells, a well-established method in cell migration analysis [47,48], including in *E. histolytica* studies [49,50]. MSD quantifies how far a cell travels from its starting point over time, reflecting migration efficiency. It provides insights into both speed and directional persistence and is typically shown in a log(MSD)-log(time) plot [47]. The slope of this plot, α, indicates the motion type: α = 2 for ballistic motion (persistent in direction), α > 1 for superdiffusion (e.g., Lévy walk, Correlated Random Walk, Fractional Brownian Motion), α = 1 for normal diffusion (Brownian motion/unbiased random movement), and α < 1 for subdiffusion (restricted movement) [47–49,51–53].

Analyses of the averaged log(MSD)-log(time) plots across all cells (Fig 3G), along with the slope α-values for individual cells from different conditions (Fig 3H), demonstrated that trophozoites incubated with *E. coli* O55 exhibited the highest α-value (α = 1.852 ± 0.009), followed by those incubated with *E. coli* K12 (α = 1.575 ± 0.055) and the control groups (e.g., α = 1.191 ± 0.039 for WT control group). This suggests that in the control groups, *E. histolytica* trophozoites exhibit movement close to normal diffusion or Brownian motion, whereas those incubated with *E. coli* O55 display enhanced superdiffusion, characterized by significantly greater directionality and speed. Notably, a subset of cells (50 out of 141) exhibited α values > 1.900, indicating a tendency toward ballistic motion. The potential contribution of stage drift during imaging, which could have artificially skewed the diffusive motion toward apparent superdiffusion, was ruled out, as it was at least two orders of magnitude smaller than the cell movements (S4 Fig).

**Fig 4:**
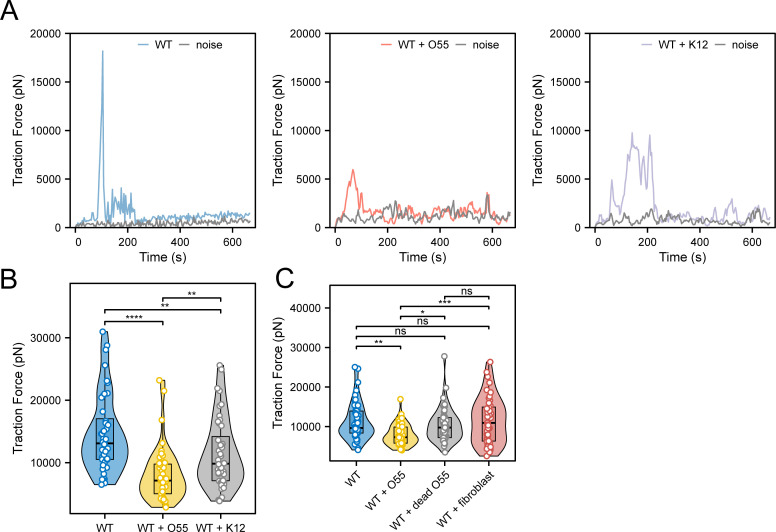
Significant difference of cellular traction force on micropillars of *E. histolytica* following short period of *E. coli* exposure. (A) Representative graphs showing the changing in traction force exerted on micropillars over time for control trophozoites (left), trophozoites incubated with enteropathogenic *E. coli* O55 (middle), and trophozoites incubated with nonpathogenic *E. coli* K12 (right), during a trophozoite passing on a micropillar. The grey curve indicates background noise of micropillars untouched by the cells. (B-C) Quantitative comparison of the max traction forces on micropillars exerted by *E. histolytica* between the different conditions: (B) WT control (n = 44), *E. coli* O55-incubated (n = 43), and *E. coli* K12-incubated (n = 44) trophozoites, as well as (C) WT control (n = 45), *E. coli* O55-incubated (n = 45), dead O55-incubated (n = 45), and fibroblast-incubated (n = 44) trophozoites. Nine cells were analyzed per condition, with 4–5 micropillars assessed per cell. Statistics, one-way ANOVA with post-hoc pairwise comparisons. * *P* < 0.05, ** *P* < 0.01, *** *P *< 0.001, **** *P *< 0.0001.

To explore whether cellular traction forces can be affected following *E. coli* exposure, we further analyzed and compared the traction forces on micropillars by *E. histolytica* under the different conditions mentioned above: WT trophozoite control, as well as trophozoites incubated with enteropathogenic *E. coli* O55, and with nonpathogenic *E. coli* K12 (Fig 4A and 4B). In a separate independent experiment, we additionally included comparisons of the traction forces of trophozoites incubated with dead *E. coli* O55 and with fibroblasts (Fig 4C). This revealed that trophozoites incubated with live *E. coli* O55 exhibited the lowest traction forces, followed by those incubated with *E. coli* K12, whereas the control groups (including WT alone, WT with dead *E. coli* O55, and WT with fibroblast co-incubation) displayed higher traction forces on the micropillar-arrays (Fig 4).

The trend in cellular traction forces and that in motility characteristics across the different *E. histolytica*-*E. coli* co-incubation conditions were opposite, prompting further consideration of the relationship between cellular traction forces and motility. In the group without exposure to *E. coli*, the cells exhibited non-directional exploratory behavior, actively extending pseudopods to tug on the surrounding pillars. At the same time, the cells seemed to exhibit greater adhesion, resulting in more significant deflection of micropillars (S3 Video). However, when exposed to enteropathogenic *E. coli* O55, the cells transitioned to an enhanced superdiffusive pattern (rapid and goal-directed), with a reduction in randomly exploring behavior and displaying noticeably smaller deflections of micropillars (S4 Video).

**Fig 5:**
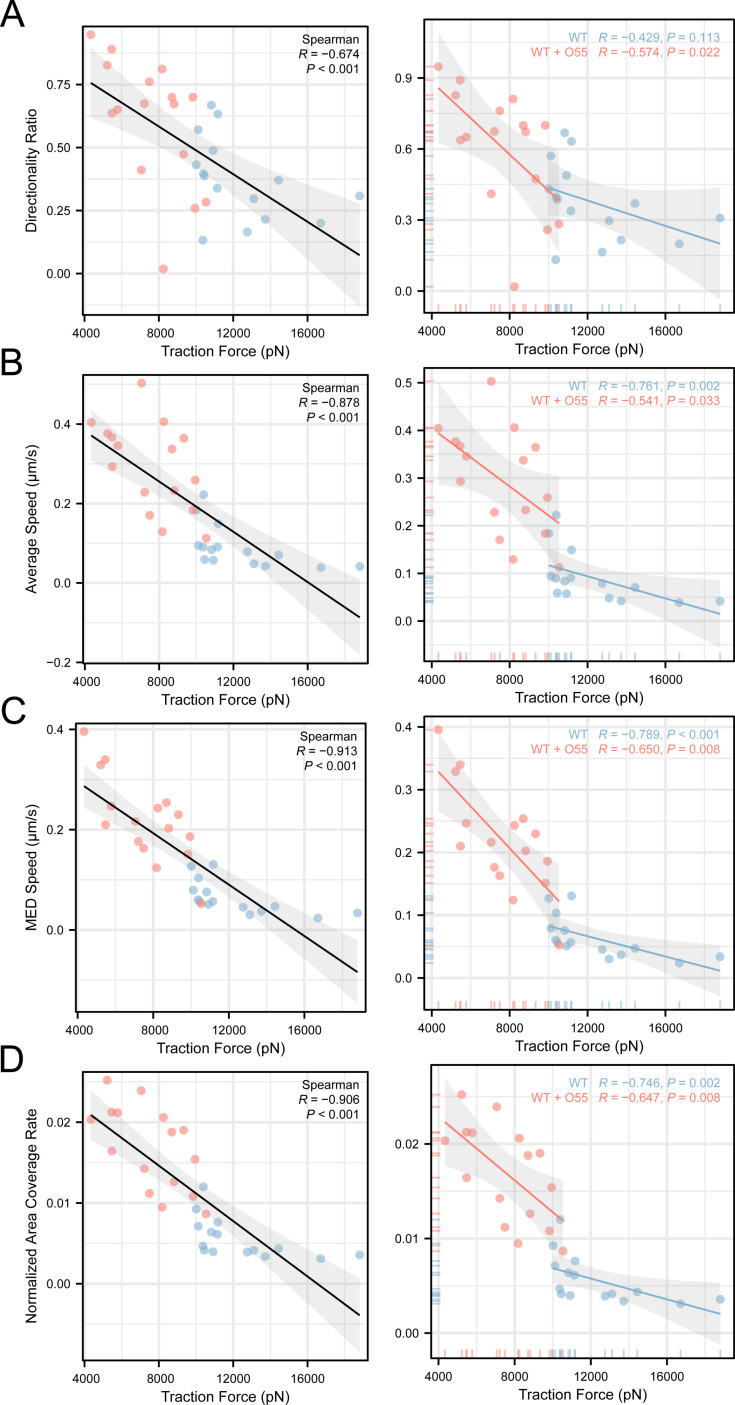
Correlation between cell motility indicators and traction force in WT control and enteropathogenic *E. coli* O55-incubated *E. histolytica.* (A) Directionality Ratio, (B) Average Speed, (C) MED Speed, (D) Normalized Area Coverage Rate (by time and cell size). n = 16 in WT group (blue) and 15 in WT + O55 group (red).

To illustrate the exact correlation between cell motility and traction force quantitively, linear regressions and Spearman correlations were performed for cells from WT and WT-*E. coli* O55 co-incubation conditions. This showed that all the four cell motility indicators (Directionality Ratio, Average Speed, MED Speed, and Normalized Area Coverage Rate) were negatively correlated with traction force (Fig 5).

### Exposure to enteropathogenic *E. coli* altered the F-actin morphology and spatial localization in *E. histolytica*

**Fig 6:**
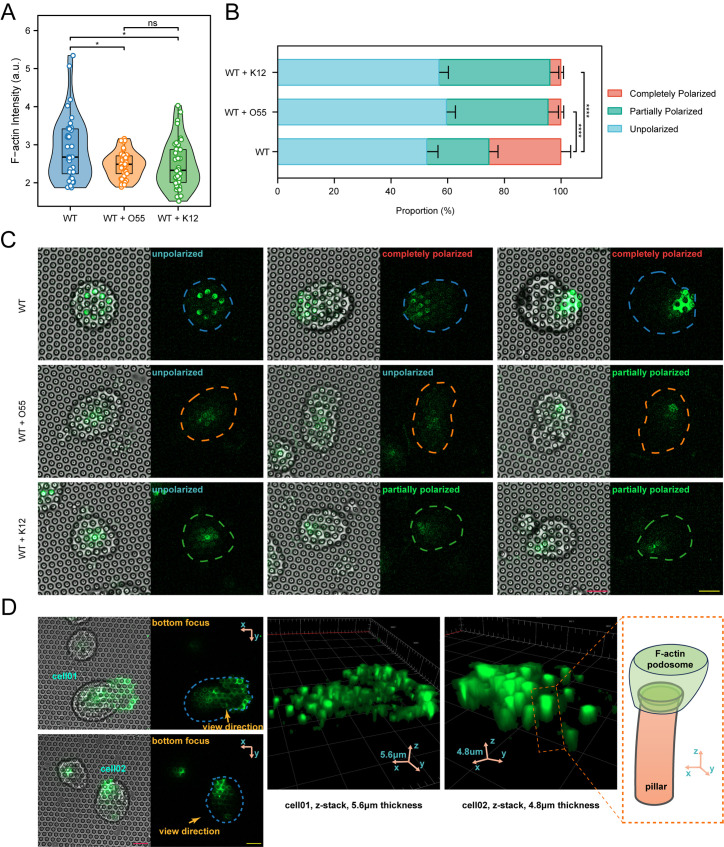
Alteration of F-actin morphology in *E. histolytica* following *E. coli* short-period exposure. (A) Quantitative comparison of Phalloidin intensity in WT control (n = 40), enteropathogenic *E. coli* O55-incubated (n = 41), and *E. coli* K12-incubated (n = 40) *E. histolytica* trophozoites. (B) Comparison of the proportion of F-actin-polarized cells in control (n = 160), *E. coli* O55-incubated (n = 202), and *E. coli* K12-incubated (n = 209) *E. histolytica*. (C) Representative confocal images (20x objective) showing the Phalloidin signal in *E. histolytica* on micropillars, both with polarized (partially and completely) and unpolarized samples. (D) Left panel: high-resolution confocal images (63x objective) captured by focusing on the plane of micropillar-array top surface, showing that F-actin formed rings that tightly encircle the corresponding micropillars, as well as filaments linking the rings. Right panel: z-stack reconstruction of F-actin spatial distribution near the micropillars, presenting podosome-like structure. The schematic diagram in the dashed-line frame illustrates the spatial relationship between an F-actin podosome-like structure and its corresponding micropillar. Scale bar in C and D: 10 μm. Statistical analyses in A and B, one-way ANOVA with post-hoc pairwise comparisons. * *P *< 0.05, ** *P *< 0.01, **** *P *< 0.0001.

Given that co-incubation of enteropathogenic *E. coli* led to remarkable variations in the motility and cellular traction forces of *E. histolytica*, we next sought to investigate whether the actin cytoskeleton plays a crucial role in explaining the underlying mechanism, as it is essential for both motility and cellular traction force. To explore how *E. coli* exposure affects F-actin organization in *E. histolytica*, WT control trophozoite, trophozoites incubated with *E. coli* O55, and trophozoite incubated with *E. coli* K12 were seeded on fibronectin-coated micropillars and incubated at 37°C for one hour, followed by fixation and subsequent staining with AF-488 Phalloidin (specifically binding to F-actin with high affinity). Notably, all images scanned by confocal microscopy were focused on the plane of the micropillars’ top surface. Consequently, a modest yet statistically significant reduction of F-actin intensity in *E. histolytica* following *E. coli* exposure was observed, with no significant difference found between trophozoites incubated with *E. coli* O55 and *E. coli* K12 (Fig 6A and 6C).

To provide additional evidence, we performed Phalloidin staining on cells plated in ibidi µ-Slide 8 Well plates. F-actin staining of *E. histolytica* trophozoites on fibronectin-coated substrates showed significantly higher intensity compared to those on uncoated micropillars (S5A and S5E Fig). Moreover, on the fibronectin-coated substrate, *E. histolytica* trophozoites exposed to *E. coli* O55 exhibited significantly lower F-actin intensity than those in the control group (S5C and S5E Fig), consistent with the results observed on the micropillars. These findings align with previous results, showing that *E. histolytica* trophozoites exerted greater traction forces on fibronectin-coated micropillars compared to uncoated ones (Fig 1B and 1C), as well as decreased traction forces when exposed to *E. coli* (Fig 4). The positive relation between F-actin intensity at micropillar top surface and cellular traction force suggests that elevated traction force on micropillars may be dependent on enhanced F-actin polymerization at pseudopods.

Given that the intracellular local polarization of F-actin is closely correlated with traction force on micropillars [38], we further analyzed the polarity of the Phalloidin signal in different conditions, categorizing it into completely polarized (towards the cell edge), partially polarized (off-center but not reaching the cell edge), and unpolarized (centered) F-actin distributions (Fig 6B and 6C). This analysis showed a significant reduction in the proportion of completely polarized cells when exposed to *E. coli* O55 and K12 (Fig 6B and 6C). This F-actin polarization pattern is consistent with the Phalloidin intensity comparisons between trophozoites incubated with *E. coli*.

Finally, we analyzed the spatial relationship between F-actin and its corresponding micropillars using high-resolution confocal scanning combined with the z-stack reconstruction mode. We discovered that F-actin accumulated near the micropillars, forming podosome-like adhesion structures that extend downward, wrapping around the top portion of the micropillars (Fig 6D, right panel). When observed from the plane of the micropillar’s top surface, F-actin formed rings that tightly encircle the corresponding micropillars, as well as filaments linking the rings (Fig 6D, left panel). This arrangement helps concentrate the force for gripping a micropillar, enabling more effective application of traction force to the micropillar [38].

**Fig 7:**
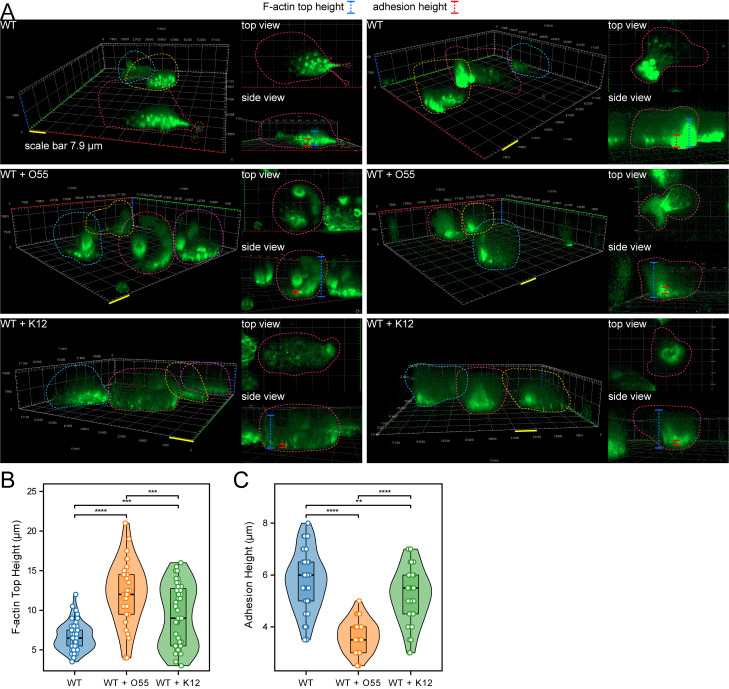
The spatial localization difference of F-actin between following *E. coli* short-period exposure. (A) Z-stack representative images showing the spatial localization differences of F-actin among three conditions: *E. histolytica* WT control trophozoites (n = 54), trophozoites incubated with enteropathogenic *E. coli* O55 (n = 32), and trophozoites incubated with *E. coli* K12 (n = 43). (B) Comparison of the height of F-actin structures in the upper part of the cell, distinct from adhesion structures on micropillars, under the different conditions. (C) Comparison of the height of F-actin adhesion structures under the different conditions. Statistical analyses in B and C, one-way ANOVA with post-hoc pairwise comparisons, ** *P* < 0.01, *** *P *< 0.001, **** *P *< 0.0001.

Next, we examined the spatial localization difference of F-actin between three conditions: *E. histolytica* WT control trophozoite, trophozoites incubated with enteropathogenic *E. coli* O55, and trophozoite incubated with *E. coli* K12, which might shed light on the mechanism regarding the negative correlation between cell locomotion and traction force on micropillars. It can be observed that without exposure to *E. coli*, F-actin primarily accumulated at podosomes (Fig 7A and 7C), possibly enabling the cells to grip the micropillars firmly and effectively. Additionally, F-actin tended to polarize toward pseudopods at the cell edge. However, upon exposure to *E. coli*, a significant amount of F-actin shifted to the upper part of the cytoplasm (Fig 7A and 7B). Consequently, the F-actin signal at the podosomes corresponding to the micropillars weakened, resulting in shorter and less prominent adhesions.

Additional analysis based on Phalloidin staining of *E. histolytica* trophozoites on fibronectin-coated ibidi µ-Slide 8 Well plates revealed that when trophozoites were incubated with *E. coli* O55, the ratio of basal surface area (determined by confocal F-actin imaging) to the total area in the bright field (non-confocal imaging) was significantly smaller compared to control trophozoites. This suggests that interaction with *E. coli* O55 reduces the contact surface area with the fibronectin-coated substrate (S5D and S5E Fig).

## Discussion

*E. histolytica*, a parasite that coexists with and feeds on bacteria in the intestinal environment, demonstrates motility closely linked to its invasiveness [6]. Despite the known short-period co-incubation of *E. coli* (O55 and K12) and *E. histolytica* trophozoites can promote *E. histolytica* cytotoxicity [45,54,55], the impact of bacterial presence on *E. histolytica*’s motility and physical forces, and its subsequent effect on invasiveness, remains underexplored.

In this study, we employed a micropillar-array system with live imaging, a technique designed for investigating traction forces on the substrate due to cellular contractility in mechanobiology [32], to explore the effect of *E. coli* (especially enteropathogenic *E. coli*) exposure on *E. histolytica* migration. Our approach involved assessing the traction forces, motility characteristics (including Directionality Ratio, Average Speed, MED Speed, and Normalized Area Coverage Rate), and F-actin spatial morphology of *E. histolytica* under conditions with and without two strains of *E. coli*. Our results indicate that *E. histolytica* modifies its physical properties and motility in response to bacterial presence, demonstrating an interplay between bacterial presence and the dynamics of the parasite’s actin-rich cytoskeleton. These findings contribute to a better understanding of the role of bacteria in modulating cellular behavior and pathogenicity.

*E. histolytica* invasion of the intestine begins with the removal of the protective mucus layer of the epithelium, facilitated by the parasite’s cysteine proteases, such as CP-A5. Following this, the trophozoites adhere to and detach enterocytes, leading to the disruption and degradation of the epithelial barrier [2]. Interestingly, the pathogenic trophozoites do not indiscriminately invade the lamina propria. Instead, they prefer to migrate along the fibronectin-rich basement membrane deep into the crypts of Lieberkühn (first stage), where they eventually penetrate the lamina propria and subsequently invade deeper structures (second stage) [2,10,56]. Using histology slides from the HPA, we highlighted that both the basement membrane and lamina propria, as well as even deeper structures of the human large intestine, are enriched with fibronectin. Therefore, in the present study, we used a fibronectin-coated substrate (micropillar arrays and ibidi plates) to mimic these pathological scenarios. In the first stage, *E. histolytica* invades parallel to the fibronectin-rich basement membrane rather than invading perpendicular to it. Thus, the invasiveness of *E. histolytica* is primarily characterized by its directional invasion along the basement membrane, allowing it to reach deep inside the crypts of Lieberkühn more rapidly. In the second stage, *E. histolytica* seeks to invade a spatially broader area as quickly as possible, ultimately leading to the formation of a flask-shaped ulcer [1]. This virulent invasion is partly mimicked in *E. histolytica*’s cytopathic activity to cause extensive destruction of the HeLa cell monolayer in a period of time. Taken together, the invasive behavior highlighted here is the rapid and directional movement of *E. histolytica* along the fibronectin-coated plane, resulting in efficient invasion to a greater distance and a broader area.

Unlike mesenchymal motility, amoeboid migration is characterized by the formation of blebs and pseudopods at the front of the trophozoite. This process, driven by hydrostatic pressure originating from the rear, involves a retraction-relaxation cycle in which the actomyosin cytoskeleton and actin-binding proteins retract the cell body and push the trophozoite forward [56–58]. Besides the role in bleb formation, F-actin is also responsible for developing adhesion structures including podosome-like, stress fiber-like structures, and adhesion plates when *E. histolytica* is placed on fibronectin substrates and interacts with fibronectin via a β1-integrin-like receptor [11–13,56].

We assume that both amoeboid migration and adhesion to fibronectin substrate contribute to the displacement of micropillars. Without exposure to *E. coli*, *E. histolytica* trophozoites exhibited non-directional exploratory behavior (localized wandering/rotating), actively using pseudopods to pull on the surrounding pillars. Further, the cells exhibited greater adhesion. This locomotion style results in more significant deflection of the micropillars. However, when exposed to enteropathogenic *E. coli*, the cells transitioned to an enhanced superdiffusive movement pattern (rapid and directional), without random exploration and active micropillar pulling, leading to a noticeably smaller deflection of the micropillars.

Given that cellular traction forces on micropillars is found to be negatively correlated with the motility (directionality, speed, and area coverage rate) of *E. histolytica* in the present study, it can be speculated that upon interaction with enteropathogenic *E. coli*, the trophozoites focus more on rapid and directional movement, thereby reducing the traction force and adhesion strength exerted on the underlying micropillars. This may be related to changes in the spatial dynamics and localization of F-actin structures.

Supporting this, we found that exposure to enteropathogenic *E. coli* significantly altered F-actin spatial distribution in *E. histolytica*. F-actin, which is primarily concentrated at podosomes and polarized toward pseudopods at the cell edge in unexposed cells, shifted towards the upper cytoplasm upon bacterial exposure, resulting in weakened adhesion structures.

We and others have found that F-actin is highly concentrated not only at the adhesion structures on the bottom and actomyosin cytoskeleton cortex at the periphery, but also in the middle of the cell [57]. Upon exposure to *E. coli*, it is possible that the shift of F-actin towards the upper part of the cytoplasm is linked to phagocytosis, potentially contributing to phagosome formation. Cell motility and phagocytosis are widely recognized as linked processes [6,59], sharing common features in mechanics and morphology, as well as key elements in biological process and cellular component, including actin, Myosin IB, Myosin II, and PAK [6,59–63]. Indeed, motility and phagocytosis are likely intertwined processes that evolved in tandem, reflecting an inseparable function. The ability to actively move towards bacterial prey would have provided a significant evolutionary advantage over passively waiting for food to arrive, enhancing competition for ingestion [59].

Hence, the F-actin structure that shifted to the upper part of the cytoplasm during *E. coli* exposure is likely involved in the rapid and direction-persistent amoeboid migration of *E. histolytica*, which is not necessarily driven by cellular active traction force on the substrate but primarily by hydrostatic pressure generated by the rapid contraction of actomyosin machinery at the rear [56,64,65]. In contrast, without *E. coli* exposure, the F-actin podosome near the micropillar surface is more dominant and primarily responsible for deflecting the pillars, and, together with the less dominant hydrostatic-powered blebs, contributes to the exploratory motility style.

There are several explanations to the negative correlation between traction forces on micropillars and the motility (directionality and speed) of *E. histolytica*. First, the enhanced superdiffusion movement requires a large accumulation of F-actin at the rear, which competes with the F-actin used in the construction of podosomes. Remarkably, the motility enhancement is powered by live enteropathogenic *E. coli* exposure, which may provide targets for cells to pursue. Phagocytosis might contribute to the shift of F-actin towards the upper part of the cytoplasm, as simply reducing cell adhesion without fibronectin coating does not enhance motility behavior. Second, the rapid and directional movement results in less time spent on a single pillar, making it difficult to form stable podosomes, thus reducing the cell’s adhesion to micropillars. Third, from a kinematic perspective: the driving force (*F*) of forward movement arises from the force and counterforce generated by the rapid and directional motion, while the resistance (*f*) stems from the adhesion between the cell and micropillars. When maximum speed and power are reached, *P*_*max*_* = F· v*_*max*_, while *F = f*. Hence, to actively increase invasion efficiency and enhance *v*_*max*_, *f* needs to be decreased, leading to reducing micropillar deformation, which is achieved by weakening adhesion, as well as reducing the contact area on the substrate.

A recently published study by Manich et al. [56] explored the impact of fibronectin on *E. histolytica* trophozoite motility, with a focus on fibronectin-induced changes in morphodynamics and an increase in adhesion force. This is consistent with our findings that fibronectin-coating enhances cell’s traction force on micropillars, which is dependent on a corresponding strengthen of cell adhesion, as evidenced by enhanced F-actin intensity in the bottom cell plane under fibronectin condition (micropillar array or ibidi plate). Manich et al. [56] also reported significantly faster migration on glass compared to fibronectin-coated surfaces, which contrasts with our findings on micropillar arrays (without live bacteria exposure), where cell motility is slightly enhanced on fibronectin-coated compared to non-coated condition. This difference could be attributed to the varying rigidity of the surfaces: glass is perceived as infinitely stiff by the cells, whereas the micropillars are several orders of magnitude softer, better mimicking physiological stiffness. Additionally, the topography of micropillars and glass is distinct. Therefore, the resistance to cell forward movement may differ between micropillar arrays and flat plates, which is also reflected in the adhesion morphology. In the study by Manich et al. [56], it is possible that cells were undergoing a “gliding” mode on the glass without fibronectin due to poor adhesion, whereas on the micropillars, cells may have been in a “searching” mode without fibronectin but switched to a migratory mode once adhesion was allowed on the fibronectin-coated surfaces. Based on the above findings, we further demonstrate that exposure to enteropathogenic *E. coli* makes *E. histolytica* reduce the adhesion and traction force on fibronectin-coated micropillars, while inducing and enhancing the superdiffusive migration.

It is of great value to further investigate the mechanism regarding what interaction occurs between *E. histolytica* and enteropathogenic *E. coli*, driving a motility enhancement. We speculate that this may be due to the *E. histolytica* chasing and engulfing bacteria, which expands the *E. histolytica*’s invasion range. Moreover, this process depends on live bacteria, suggesting that the molecular interactions between *E. histolytica* and enteropathogenic *E. coli* rely on bacterial molecules that are only present or functional in living bacteria.

In the natural world, superdiffusive movement patterns (such as Lévy walks, Correlated Random Walk, etc.) are commonly adopted by animals [48,66], with Lévy walks being a common strategy among various animal predators searching for scarce prey or food sources, suggesting an evolutionary advantage in employing such movement patterns [48]. In the microscopic world, superdiffusive movement patterns are also utilized by various cells and microorganisms, such as cancer cells [48], immune cells [67,68], stem cells [69], epithelial cells [70,71], and bacteria [72–74]. These include Lévy walks [48,67,72,73], Correlated Random Walk [68,71], Fractional Brownian Motion [68,71], etc. These movement patterns are likely crucial for their survival, nutrient acquisition, proliferation, and dissemination in complex physiological or pathological microenvironments.

An instructive kinematic study on cancer metastasis demonstrates that the trajectories of metastatic cancer cells exhibit enhanced Lévy-like movement patterns, while non-metastatic cancer cells move more diffusively. This migration mode might provide metastatic cells with an effective strategy for disseminating and searching suitable sites to establish new metastatic sites, which is a similar goal for *E. histolytica* [48].

We co-incubated *E. histolytica* with two strains of *E. coli*, but the outcomes differed between the two strains. O55 is classified as an enteropathogenic *E. coli* [75,76]. However, K12 is not enteropathogenic, but a laboratory strain that has undergone extensive mutagenesis, leading to the loss of several genetic traits associated with virulence [77,78]. *E. histolytica* co-incubated with O55 not only significantly increased *E. histolytica*’s destruction of cultured HeLa cell monolayers, but also markedly enhanced superdiffusion movement, while significantly reducing its traction force on the fibronectin-rich substrate. In contrast, the cytopathic activity of *E. histolytica* and the associated changes in motility and cellular mechanics were less pronounced when co-incubated with K12 (compared to O55). These findings highlight a clear link between the parasite’s motility/traction force and cytopathic activities, suggesting that changes in kinematics and mechanics play a pivotal role in the virulence and pathogenicity of *E. histolytica*. It is reported that the galactose and N-acetylgalactosamine (Gal/GalNAc) residues on the surface lipopolysaccharide of O55 can be recognized by and interact with *E. histolytica*’s Gal/GalNAc lectin [79,80]. In contrast, the interaction between K12 and *E. histolytica* may rely on different mechanisms, such as type 1 fimbriae mediated binding to mannose-containing receptors on the *E. histolytica* surface [79,81]. These distinct molecular interactions may influence F-actin cytoskeleton-associated cellular components and pathways, thus affecting the locomotion, mechanics, and phagocytosis of *E. histolytica.*

**Fig 8:**
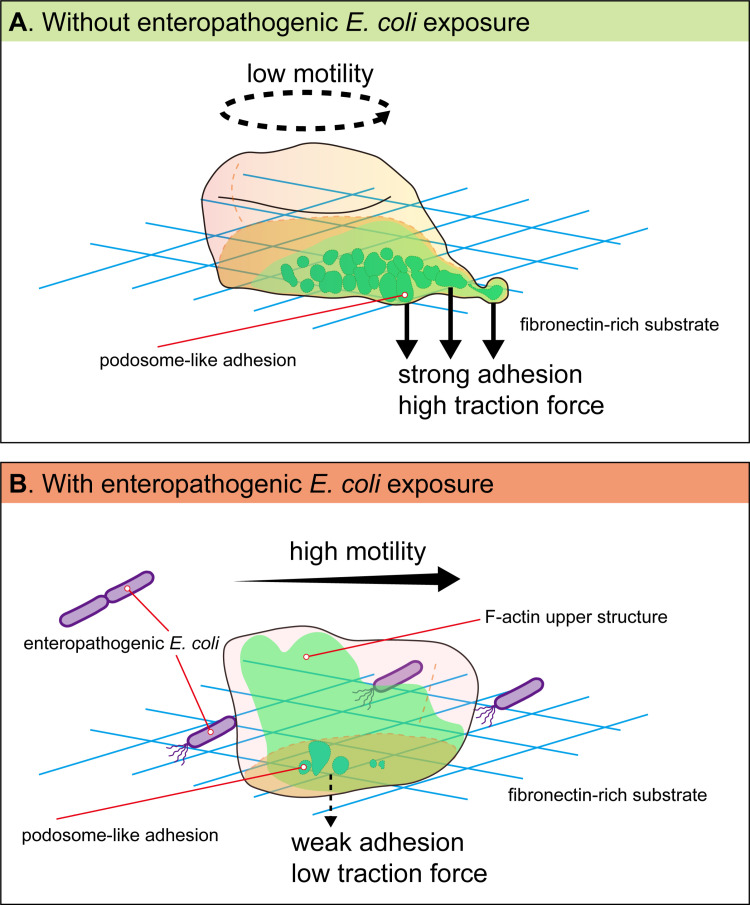
A hypothetical model illustrating the alteration in motility and traction force on a fibronectin-rich substrate during *E. histolytica* trophozoite migration with and without exposure to enteropathogenic *E. coli.* (A) Without enteropathogenic *E. coli* exposure, the *E. histolytica* trophozoites exhibit a non-directional exploratory behavior, actively using pseudopods to tug on the surrounding fibronectin-rich substrate. F-actin primarily accumulates at podosome-like adhesion structures extending downward, which are notably large, elevated and strong, possibly enabling the cells to grip the substrate firmly and effectively. Additionally, F-actin polarizes toward pseudopods at the cell edge, exerting high traction force to the fibronectin-rich substrate. (B) Co-incubation with live enteropathogenic *E. coli* enhances *E. histolytica* trophozoites’ motility, characterized by enhanced superdiffusion movement patterns (increased directionality, speed, and area coverage rate). This increased motility is accompanied by a reduction or weakening of F-actin podosome-like adhesion structures and their cell-edge polarization, as well as a decrease in traction forces on the fibronectin-rich substrate. At the same time, there is increased F-actin localization in the upper part of the cytoplasm, which may be linked to the active motility of the trophozoites. This locomotion leads to rapid and broader dissemination, which can promote the invasiveness of *E. histolytica*.

In conclusion, short-term co-incubation with live enteropathogenic *E. coli* enhances *E. histolytica*’s superdiffusion movement, characterized by increased directionality, speed, and area coverage rate. This activated motility is accompanied by a reduction in F-actin-dependent traction forces and podosome-like structures on the fibronectin-rich substrate, but with increased F-actin localization in the upper part of the cytoplasm. This locomotion leads to rapid and broader dissemination, which can promote the invasiveness of *E. histolytica* (Fig 8). This study provides new insights into the role of physical behaviors in the virulence of *E. histolytica* and offers new perspectives for the clinical prevention and treatment of amebiasis.

## Materials and methods

### *E. histolytica* strains

The *E. histolytica* strain HM-1:IMSS was graciously supplied by Prof. Samudrala Gourinath from Jawaharlal Nehru University, New Delhi, India. Trophozoites of *E. histolytica* strain HM-1:IMSS was cultured at 37°C in 13 × 100 mm screw-capped Pyrex glass tubes containing Diamond’s TYI-S-33 medium until they reached the exponential growth phase. Trophozoites were then harvested from the culture tubes using the previously outlined method of tapping followed by centrifugation, as detailed in prior protocols [82].

### Bacterial strains

The enteropathogenic *E. coli* strain TW04062 O55 is from the Thomas S. Whittam STEC Center. The nonpathogenic *E. coli* strain MG1655 K12 was provided by Sima Yaron from the Department of Biotechnology and Food Engineering at the Technion. *E. coli* cultures were incubated at 37°C in Luria-Bertani (LB) medium.

### Preparation of dead bacteria

A total of 1 × 10^9 bacteria were mixed with 1 mL of 4% formaldehyde (buffered to pH 7.2–7.4) and incubated at room temperature for 30 minutes. The bacteria were then centrifuged, washed once with PBS, followed by three washes with 0.1 M glycine, and finally washed once with TYI medium before resuspension.

### Measurement of cytopathic activity

The assessment of trophozoites’ destruction of cultured HeLa cell monolayers was conducted utilizing a previously outlined methodology [45]. In brief, a concentration of 1 × 10^6 trophozoites/mL was incubated with *E. coli* O55 (live or dead) or *E. coli* K12 (1 × 10^9/mL) in serum-free Diamond’s TYI-S-33 medium at 37°C for 30 minutes while shaking. Afterwards, most bacteria were removed by washing the trophozoites with TYI medium through centrifugation at 726 × g for three cycles. Then trophozoites (10^5 per well) were then seeded onto and incubated with HeLa cell monolayers (~90% confluency) in 24-well tissue culture plates with serum-free Diamond’s TYI-S-33 medium at 37°C for 60 minutes. The incubation process was stopped by transferring the plates to ice, followed by removal of unattached trophozoites through washing with cold phosphate-buffered saline (PBS). The remaining HeLa cells adhered to the plates were subsequently stained with methylene blue solution (0.1% in 0.1 M borate buffer, pH 8.7). Extraction of the dye from stained cells was performed using 0.1 M HCl, and the color intensity of the extracted dye was measured spectrophotometrically at OD660. For the bacterial-only treatment group, 5 × 10^7 of *E. coli* O55 were directly seeded onto and incubated with HeLa cell monolayers, with all other conditions kept consistent with the other groups.

### Micropillar-array system and traction force calculation

PDMS micropillar-arrays were employed in this study, which allowed us to detect and measure the traction force that cells applied on the micropillars [32,38]. Based on the microscope time-series imaging of micropillars within a particular period, we spatiotemporally tracked and recorded the location of every micropillar, which was then calculated to micropillar displacement (*δ*) and then transferred to traction force (*F*) [32] (Fig 1A).

The rigidity of PDMS micropillar-array is only altered by changing micropillar height, while the cross-sectional area and chemical properties of the micropillars remain constant. The diameter of the circular, micropillars cross-section is two μm, and the center-to-center spacing between adjacent micropillars is four μm.

Tracking of micropillars movements over time was performed with NanoTracking plugin of Fiji/ImageJ (National Institutes of Health), the detailed algorithm of which is described previously [83]. Briefly, it employed a cross-correlation technique that enabled the software to obtain the relative x and y position of every micropillars in every frame of the time-series movie in nanometer-level. We only selected for analysis the cell migration-associated micropillars that were not in contact with any cells (zero position) at the beginning of the movie. Micropillar displacement (*δ*), which is caused by traction force (*F*) when cell edge/body contact the micropillars, at each frame, is calculated based on the distance from current to the zero position. Traction force (*F*) is calculated by multiplying micropillar displacement (*δ*) by the spring constant of the corresponding pillar height (Fig 1A). Micropillar arrays with heights of 5.3, 9.4, and 13.2 μm have external rigidities (spring constant, *k*_*pillar*_) of 2, 6, and 31 pN/nm, respectively, as calculated using the Euler-Bernoulli beam theory [38]. The curve of traction force as a function of time *F(t)* of a micropillar was plotted using MATLAB (MathWorks, v2018b) with *Smooth* function, using a smoothing parameter of 0.01.

### PDMS micropillars-array fabrication and fibronectin coating

Pillar fabrication was achieved by pouring PDMS, which was mixed at 10:1 with its curing agent (SYLGARD 184, Dow Corning), over silicon molds with holes at fixed depths and geometric distance. The mold was then put upside down, onto a glass-bottom 35-mm dish (D35-20–0-N, Cellvis), which was followed by incubation at 60°C for 12 hours. After cooling to room temperature, the mold was peeled off while both the mold and the PDMS pillar cast were immersed in absolute ethanol to prevent micropillars from collapsing. Afterwards, the ethanol was replaced by PBS, and then human plasma full-length fibronectin (FC010–10MG, Merck) was added to the dish, making a coating solution with final concentration of 10 μg/mL for a one-hour surface-coating at 37°C [32]. Finally, residual fibronectin was washed away by four serial replacements with serum-free Diamond’s TYI-S-33 medium. For the experiments using micropillars without fibronectin coating, absolute ethanol was replaced by PBS, which was then replaced by serum-free Diamond’s TYI-S-33 medium.

### Cell seeding on micropillar-arrays and time-series imaging

A total of 1 × 10^5 *E. histolytica* trophozoites were collected through tapping and subsequent centrifugation. These trophozoites were then seeded onto a fibronectin-coated (or non-coated) micropillar-array in a 35 mm glass-bottom dish filled with serum-free Diamond’s TYI-S-33 medium. The system was incubated in a heating chamber at 37°C located on Zeiss LSM800 confocal microscope. Regarding *E. histolytica* trophozoites treated with bacteria, a concentration of 1 × 10^6 trophozoites/mL was incubated with *E. coli* O55 (live or dead) or *E. coli* K12 (1 × 10^9/mL) in serum-free Diamond’s TYI-S-33 medium at 37°C for 30 minutes while shaking. Afterwards, most bacteria were removed by washing the trophozoites with TYI medium through centrifugation at 726 × g for three cycles. The trophozoites were then resuspended in TYI medium, followed by seeding (1 × 10^5 trophozoites) onto a micropillar-array described above. Regarding *E. histolytica* trophozoites treated with fibroblasts, 1 million cells were pre-seeded per well in a six-well plate for two hours before adding 0.5 million *E. histolytica* trophozoites. Co-incubation was carried out in serum-free Diamond’s TYI-S-33 medium at 37°C with 5% CO₂ for 30 minutes. The trophozoites were then collected by pipetting, centrifuged, washed once with TYI medium, and resuspended in TYI medium. Subsequently, 1 × 10^5 trophozoites were seeded onto a micropillar array as described above. Bright-field time-lapse imaging for *E. histolytica* on micropillars was performed using a 20 × objective at 37°C. Images were scanned every four seconds with a time-series mode. Fibroblasts (mouse embryonic fibroblasts) used in this study is a gift from M. Sheetz (MBI Singapore and the University of Texas Medical Branch) [84].

### Phalloidin staining on micropillars

A concentration of 1 × 10^6 trophozoites/mL was incubated with or without *E. coli* O55 or *E. coli* K12 (1 × 10^9/mL) in serum-free Diamond’s TYI-S-33 medium at 37°C for 30 minutes while shaking. Afterwards, most bacteria were removed by washing the trophozoites with TYI medium through centrifugation at 726 × g for three cycles. The trophozoites were then resuspended in TYI medium, followed by seeding onto a micropillar-array and incubated at 37°C for an hour. Then trophozoites were fixed by 4% paraformaldehyde (PFA) for 15 minutes at room temperature. After washing away PFA by PBS, the cells were permeabilized with 0.2% Triton-X for 10 minutes at room temperature. Afterwards, the cells were stained with Alexa Fluor 488 Phalloidin (A12379, Invitrogen) with a dilution of 1:1000 for 15 minutes at room temperature. After another PBS wash, the cells were kept in PBS solution with SlowFade Gold Antifade Mountant (#S36937, Invitrogen), and then imaged by Zeiss LSM800 confocal microscope with a magnification of 20x with Airyscan processing.

Regarding three-dimensional reconstruction of F-actin structures, the cell is imaged with 63x objective scanned with Airyscan and Z-stack mode (0.2 µm interval between neighboring slices with a total thickness around 5 µm), followed by Airyscan processing and deconvolution.

### Cell motility analysis

The cell motility analysis begins with converting the time-series imaging in CZI format to 8-bit image stacks (TIF format) in Fiji. The observed experimental domain measures 266.21 × 266.21 μm, with a resolution of 0.144 μm per pixel.

To measure and evaluate stage drift of imaging (S4 Fig), five randomly selected micropillars, unaffected by any disturbances, were used for nano-tracking. Drift in both the x and y directions was calculated for each frame. Within a complete video of approximately 300 frames (4 seconds per frame), the drift is within 250 nm, which is less than 2 pixels, far smaller than the size of the cells and the distance the cells migrate within 300 frames. To note, the average cell size is about 481.88 ± 6.65μm^2^ in area and 24.75 ± 0.17 μm in diameter.

To obtain the mask of every cell in each frame of time-series image stacks, we developed a sophisticated cell segmentation and tracking framework that harnesses the zero-shot generalization capabilities of the Segment Anything Model 2.1 Large (SAM 2.1 Large) (https://ai.meta.com/sam2/). Our approach implements a novel double segmentation methodology designed to overcome persistent challenges in cell segmentation and tracking, including stability issues, contrast-dependent segmentation errors, rapid morphological changes, and efficiency degradation when tracking multiple objects simultaneously.

Our framework employs two sequential segmentation phases for each frame, implemented in Python 3.12 with the SAM 2.1 Large model serving as the foundation for both phases. The first phase utilizes the SAM 2.1 model in high sensitivity mode to detect new objects entering the Field of View (FOV) and to update the positions of previously tracked objects. The second phase employs bounding-box based segmentation in SAM 2.1 to achieve high accuracy cell delineation. This dual approach effectively addresses low contrast conditions and mitigates accuracy loss in cell segmentation, particularly at cell boundaries.

Although cells exhibit rapid morphological changes, their inter-frame movement remains relatively slower, enabling the implementation of similarity-based cell tracking. For comprehensive analysis, we monitor multiple parameters for each cell, including centroid position, intensity, area, and intercellular interactions. These parameters are systematically recorded alongside annotated videos and cell masks to facilitate thorough analysis and validation. The examples of cell tracking with cell contours for all groups is shown in S1 Appendix.

By using the cell masks, the position (x,y) coordinate and area of each cell at every frame was calculated and recorded by *scikit-image package (version 0.23.2)* in Python 3.12. Trajectory plots for cells in an experimental group are generated by R4.4.0 with *ggplot2*, with the coordinates of the cells’ starting points transformed to position (0,0).

The time duration of the recorded trajectories for the WT control group had a median of 1030 seconds, with an interquartile range (IQR) of 608–1332 seconds. For the trophozoite incubated with the O55 group, the median was 324 seconds, with an IQR of 240–472 seconds. For the other groups, the median times were as follows: WT on micropillars without fibronectin-coating: 1060 seconds (IQR: 968–1116), WT + K12: 560 seconds (IQR: 360–1152), WT + dead O55: 640 seconds (IQR: 544–858), and WT + fibroblast: 740 seconds (IQR: 664–1160). There is no truncation in the data, as consecutive frames are recorded at 4-second intervals. Recording stops when any part of the cell moves out of the frame. Additionally, we ensured that the extracted cell trajectories did not involve any obvious cell contact or collision.

The motility parameters used in this study are listed below and were calculated in R4.4.0. The schematic diagram in (Fig 3F) illustrates the related definitions.

1. Average Speed: The total accumulated trajectory length divided by time:


$$\begin{array}{c} \text{Average\textbackslash{} Speed}=\frac{\sum_{i=1}^{N-1}d_{i}}{T} \end{array}$$ (1)

where *d*_*i*_ represents the Euclidean distance between consecutive time points, and *T* is the total duration of the experiment. To calculate the Average Speed, the coordinates and trajectory of each cell are smoothed using loess with a span of 0.3.

2. Maximum Euclidean Displacement (MED) Speed: The maximum Euclidean displacement divided by the corresponding time interval:


$$\begin{array}{c} \text{MED\textbackslash{} Speed}=\frac{\text{max}(\sqrt{\left\{{\left({\text{x}}_{\text{t}}-{\text{x}}_{0}\right)}^{2}+{\left({\text{y}}_{\text{t}}-{\text{y}}_{0}\right)}^{2}\right\}})}{\left(\text{t}-{\text{t}}_{0}\right)} \end{array}$$ (2)

where (*x*_*0*_*, y*_*0*_) is the starting position, (*x*_*t*_*, y*_*t*_) is the position corresponding to the MED, and (*t - t*_*0*_) is the time interval.

3. Directionality Ratio: The ratio of Euclidean distance to accumulated trajectory length:


$$\begin{array}{c} \text{Directionality\textbackslash{} Ratio}=\frac{\sqrt{\left\{{\left({\text{x}}_{N}-{\text{x}}_{0}\right)}^{2}+{\left({\text{\textbackslash{}text y}}_{\text{N}}-{\text{y}}_{0}\right)}^{2}\right\}}}{\sum_{i=1}^{N-1}d_{i}} \end{array}$$ (3)

where the numerator represents the Euclidean distance from the start to the endpoint, and the denominator is the total accumulated trajectory length. A value closer to 1 indicates more directed movement.

4. Normalized Area Coverage Rate: The accumulated covered area normalized by both time and cell size:


$$\begin{array}{c} \text{Normalized\textbackslash{} Area\textbackslash{} Coverage\textbackslash{} Rate}=\frac{\text{Covered\textbackslash{} Area}}{\text{T}\times \text{Mean\textbackslash{} Cell\textbackslash{} Area}} \end{array}$$ (4)

where *Covered Area* represents the total accumulated area explored by the cell during its migration (with cell overlapping regions not counted multiple times), *T* is the total time, and *Mean Cell Area* is the average cell size. This metric reflects the cell’s efficiency in exploring space relative to its size and time.

### Inhibitors treatment

To assess the effects of different inhibitors on *E. histolytica* traction force and migration, *E. histolytica* trophozoites were pre-incubated with E64d (10 µM for 16 hours), metronidazole (MNZ, 5 µM for 16 hours), or Cytochalasin D (CytD, 5 µM for 24 hours) at 37°C. Control trophozoites were treated with DMSO. A total of 1 × 10^5 trophozoites were collected via tapping and subsequent centrifugation. These trophozoites were then seeded onto a fibronectin-coated micropillar-array in a glass-bottom dish filled with serum-free Diamond’s TYI-S-33 medium, supplemented with the same concentration of inhibitors as used during pre-incubation, followed by live-imaging on a confocal microscope.

### Phalloidin staining on ibidi µ-Slide 8 Well plates

A total of 2 × 10^5 *E. histolytica t*rophozoites were collected via tapping and subsequent centrifugation. These trophozoites were then seeded onto an ibidi µ-Slide 8 Well plate (#80826, ibidi GmbH) pre-coated with or without fibronectin and incubated in serum-free Diamond’s TYI-S-33 medium at 37°C for one hour. Regarding *E. histolytica* trophozoites treated with bacteria, a concentration of 1 × 10^6 trophozoites/mL was incubated with *E. coli* O55 or *E. coli* K12 (1 × 10^9/mL) in serum-free Diamond’s TYI-S-33 medium at 37°C for 30 minutes while shaking. Afterwards, most bacteria were removed by washing the trophozoites with TYI medium through centrifugation at 726 × g for three cycles. The trophozoites were then resuspended in TYI medium, followed by seeding onto an ibidi plate and incubated at 37°C for one hour. Next, the plates were washed three times with PBS to remove residual TYI-S-33 medium. Trophozoites were then fixed with 4% PFA in PBS for 15 minutes at room temperature. Following PFA removal by PBS washing, cells were permeabilized with 0.2% Triton X-100 for 10 minutes at room temperature. Subsequently, the cells were stained with Alexa Fluor 488 Phalloidin (A12379, Invitrogen) with a dilution of 1:1000 for 15 minutes at room temperature. Later, cells were washed with PBS and mounted with SlowFade Gold Antifade Mountant. Imaging was performed using confocal microscope with a 63x objective and Airyscan processing.

### Image segmentation and polarization categorization

For the planar confocal imaging for fixed cells, all images included in the same analysis were obtained under identical experimental conditions and scanning parameters. The fluorescence signal range and cell contours were delineated using the SAM model (Segment Anything Model, https://segment-anything.com/) to generate the corresponding masks. These masks were then analyzed in Fiji for area, intensity, and center of mass coordinates.

For the analysis of F-actin adhesion polarization on micropillars, the following criteria were applied: 1) If the adhesion region overlapped with the cell boundary, the cell was categorized as completely polarized. 2) For the remaining cells, if the cell’s center of mass was within the adhesion region, the cell was categorized as unpolarized. 3) If the cell’s center of mass was outside the adhesion region, the cell was categorized as partially polarized.

### Statistical analysis

Statistical analyses were conducted utilizing both Prism 9 (Graphpad Software Inc.) and *R* version *4.4.0*. Plots are generated by *ggplot2* and *ggpubr* packages with *R* version *4.4.0*. Unless otherwise specified, significance was assessed using one-way ANOVA for multiple comparisons (with Holm-Sidak method for *post-hoc* pairwise comparisons) and unpaired two-tailed t-test for two-group comparisons. For data that did not meet normality assumptions, corresponding nonparametric methods, including Kruskal-Wallis (with Dunn’s test for post-hoc pairwise comparisons) and Mann-Whitney U tests, were used. Spearman correlation analyses were performed using *ggpubr* package. Chi-square test was performed by *gmodels* and *tableone* packages.

## Supporting information

S1 Fig Fibronectin expression in human large intestinal mucosa. (A) Fibronectin expression in colon mucosa. (B) Fibronectin expression in rectum mucosa. Fibronectin is prominently expressed in the subepithelial basement membrane (indicated by arrows), the lamina propria, and deeper structures. Images are sourced from the Human Protein Atlas (HPA) database (www.proteinatlas.org). (C) Western blotting results sourced from Atlas Antibodies (www.atlasantibodies.com) demonstrate the specificity of the antibody (HPA027066) used for staining the tissues shown in A and B.
(TIF)

S2 Fig Motility properties of *E. histolytica* show slightly yet statistically significant promoted motility on fibronectin-coated micropillars than on uncoated ones. (A) Trajectory plots of trophozoites on fibronectin (FN)-coated (right panels, n = 82) and uncoated (left panels, n = 61) micropillars, with color mapping indicating time (top row) and the average speed over every four seconds between two consecutive frames (bottom row). (B-D) Quantitative comparisons of Directionality Ratio (B), Average Speed (C), MED speed (D), and Normalized Area Coverage Rate (by time and cell size) (E) of *E. histolytica* between migrating on fibronectin-coated and uncoated micropillars, with Mann-Whitney U tests. (F) Comparison of the slopes (α) of the fitted lines for the log(MSD)-log(time) plot (Fig 3G) representing individual cell migration under different conditions: fibronectin-coated (n = 82) versus uncoated micropillars (n = 61), with Mann-Whitney U test. * *p *< 0.05, ** *P* < 0.01.
(TIF)

S3 Fig Trajectory plots of trophozoites migration under different conditions: WT control (n = 82), incubation with enteropathogenic *E. coli* O55 (n = 141), incubation with nonpathogenic *E. coli* K12 (n = 41), incubation with dead *E. coli* O55 (n = 79), as well as incubation with fibroblasts (n = 37), with color mapping indicating the average speed over every four seconds (between two consecutive frames). Noteworthily, O55 appears more frequently in cyan, blue, and purple (corresponding to higher speeds), while the other groups predominantly show red and yellow, clearly indicating that the O55 group moves faster.
(TIF)

S4 Fig Evaluation of stage drift of live imaging. Five randomly selected micropillars, unaffected by any disturbances, were used for nano-tracking. Drift in both the x and y directions was calculated for each frame. Two examples of the drift for each the WT and WT + O55 groups in the x and y directions was illustrated. It can be observed that within a complete video of approximately 300 frames (4 seconds per frame), the drift is within 250 nm, which is less than 2 pixels, far smaller than the size of the cells and the distance the cells migrate within 300 frames. Note: resolution 0.144 μm/pixel, average cell size is 481.88 ± 6.65μm^2^ in area and 24.75 ± 0.17 μm in diameter.
(TIF)

S5 Fig Alteration of F-actin morphology in *E. histolytica* migrating on ibidi µ-Slide 8 Well plates in different conditions. (A) Normalized Phalloidin intensity was quantitatively compared between *E. histolytica* trophozoites migrating on surface with (n = 77) and without (n = 28) fibronectin (FN) coating. (B) The normalized ratio of basal surface area (determined by confocal F-actin imaging) to the total area in the bright field (non-confocal imaging) was compared between trophozoites under two conditions of (A). (C) Phalloidin intensity was quantitatively compared among *E. histolytica* trophozoites under three conditions: control (n = 77), incubated with *E. coli* O55 (n = 67), and incubated with *E. coli* K12 (n = 61), on fibronectin (FN)-coated ibidi plates. (D) The normalized ratio of basal surface area (determined by confocal F-actin imaging) to the total area in the bright field (non-confocal imaging) was compared between trophozoites under three conditions of (C). (E) Representative images illustrate the differences in features compared in A-D. The yellow line outlines the basal surface area (determined by confocal F-actin imaging) of the cell. Statistical analyses, one-way ANOVA with post-hoc pairwise comparisons. * *P *< 0.05, ** *P* < 0.01, *** *P* < 0.001, **** *P* < 0.0001.
(TIF)

S1 Video A representative video showing the motility of *E. histolytica* trophozoites on fibronectin-coated micropillar array, without exposure to *E. coli*. Time stamp (minutes: seconds). (AVI)

S2 Video A representative video showing the motility of *E. histolytica* trophozoites incubated with *E. coli* O55 on fibronectin-coated micropillar array. Time stamp (minutes: seconds). In the videos, the curved organisms filling the screen are actually shadows of *E. coli*, distributed at different heights, due to the non-confocal nature of live imaging. In reality, the number of bacteria directly in contact with *E. histolytica* trophozoites is limited.
(AVI)

S3 Video A representative video cropped from S1 Video shows pillar displacement as an *E. histolytica* trophozoite migrates on a fibronectin-coated micropillar array, without *E. coli* exposure. Time stamp (minutes: seconds). The trophozoite exhibits a non-directional exploratory behavior, actively using pseudopods to tug on the surrounding pillars. At the same time, the trophozoite seems to exhibit greater adhesion, resulting in more significant deflection of the micropillars.
(AVI)

S4 Video A representative video cropped from S2 Video shows pillar displacement as an *E. histolytica* trophozoite migrates on a fibronectin-coated micropillar array, incubated with *E. coli* O55. Time stamp (minutes: seconds). When exposed to *E. coli* O55, the trophozoite transitions to rapid, goal-directed movement without actively pulling on the micropillars or engaging in random exploration, resulting in noticeably smaller micropillar deflections. In the videos, the curved organisms filling the screen are actually shadows of *E. coli*, distributed at different heights, due to the non-confocal nature of live imaging. In reality, the number of bacteria directly in contact with *E. histolytica* trophozoites is limited.
(AVI)

S1 Appendix Examples of AI-based cell tracking with cell contours for all the experimental groups. (ZIP)
