# Correction: Enteropathogenic *Escherichia coli* induces *Entamoeba histolytica* superdiffusion movement on fibronectin by reducing traction forces

**DOI:** 10.1371/journal.ppat.1013960

**Published:** 2026-02-23

**Authors:** Yuanning Guo, Jun Ye, Ariel Shemesh, Anas Odeh, Meirav Trebicz-Geffen, Haguy Wolfenson, Serge Ankri

The raw data underlying Figs 1–7, S2, and S5 were not originally provided with this article [1]. The authors have provided the data as Supporting Information file S1 File.

With this Correction, all relevant data are now provided in the below Supporting Information file.

Additionally, the ORCID iDs are missing for the co-first authors. Please see the authors’ respective ORCID iDs here:

- Author Yuanning Guo’s ORCID iD is: 0000-0002-1391-0914 (https://orcid.org/0000-0002-1391-0914).
- Author Jun Ye’s ORCID iD is: 0000-0002-8240-9146 (https://orcid.org/0000-0002-8240-9146).

## Supporting information

S1 File Raw data underlying Figs 1-7, S2, and S5. (ZIP)
