# Supplementary figures and images for: Enteropathogenic Escherichia coli induces Entamoeba histolytica superdiffusion movement on fibronectin by reducing traction forces
Source: PLoS Pathog. 2025 May 23;21(5):e1012618. doi: 10.1371/journal.ppat.1012618 (PMC12140418; doi:10.1371/journal.ppat.1012618)

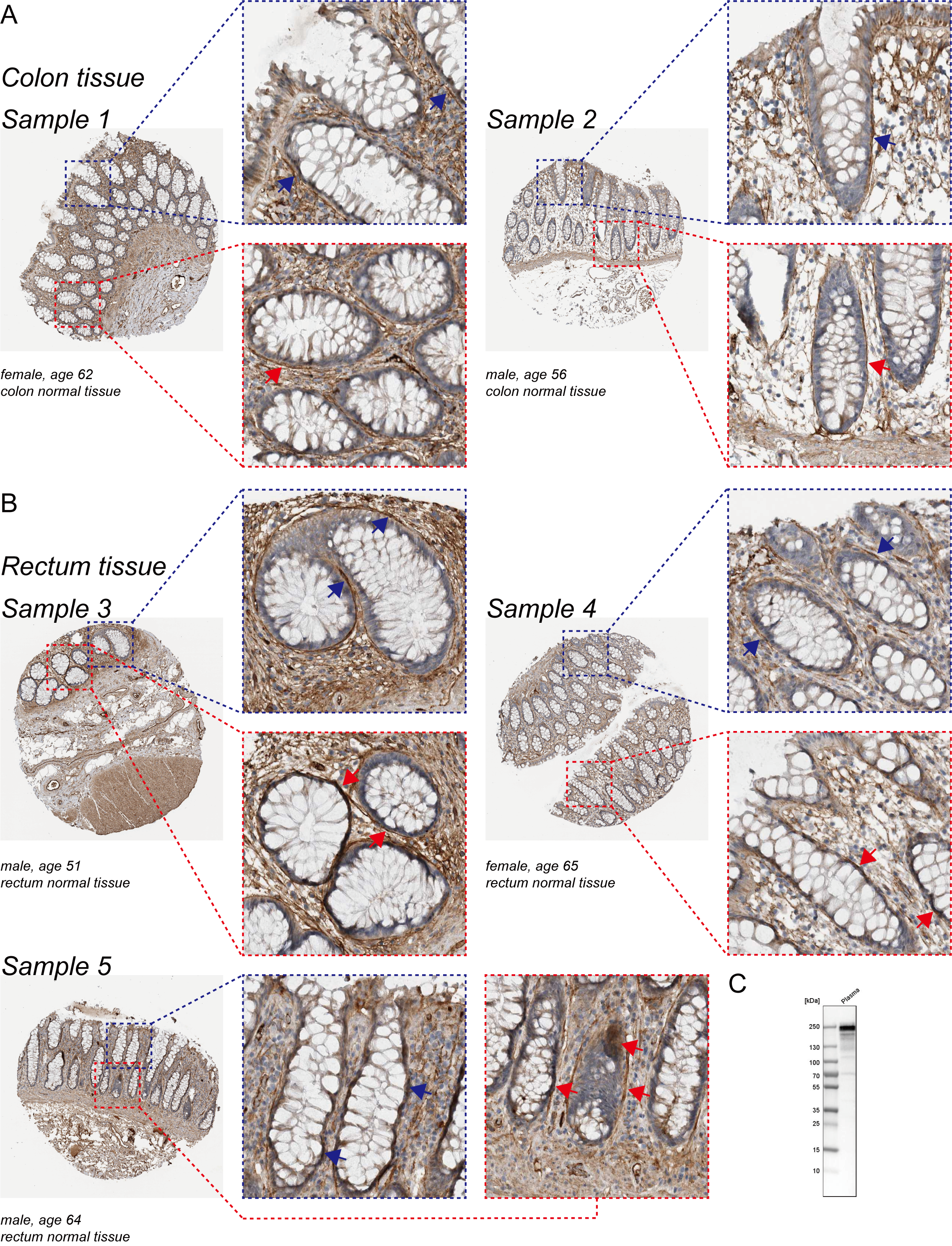

Supplement: S1 Fig — (A) Fibronectin expression in colon mucosa. (B) Fibronectin expression in rectum mucosa. Fibronectin is prominently expressed in the subepithelial basement membrane (indicated by arrows), the lamina propria, and deeper structures. Images are sourced from the Human Protein Atlas (HPA) database (www.proteinatlas.org). (C) Western blotting results sourced from Atlas Antibodies (www.atlasantibodies.com) demonstrate the specificity of the antibody (HPA027066) used for staining the tissues shown in A and B. (TIF) [file ppat.1012618.s001.tif]

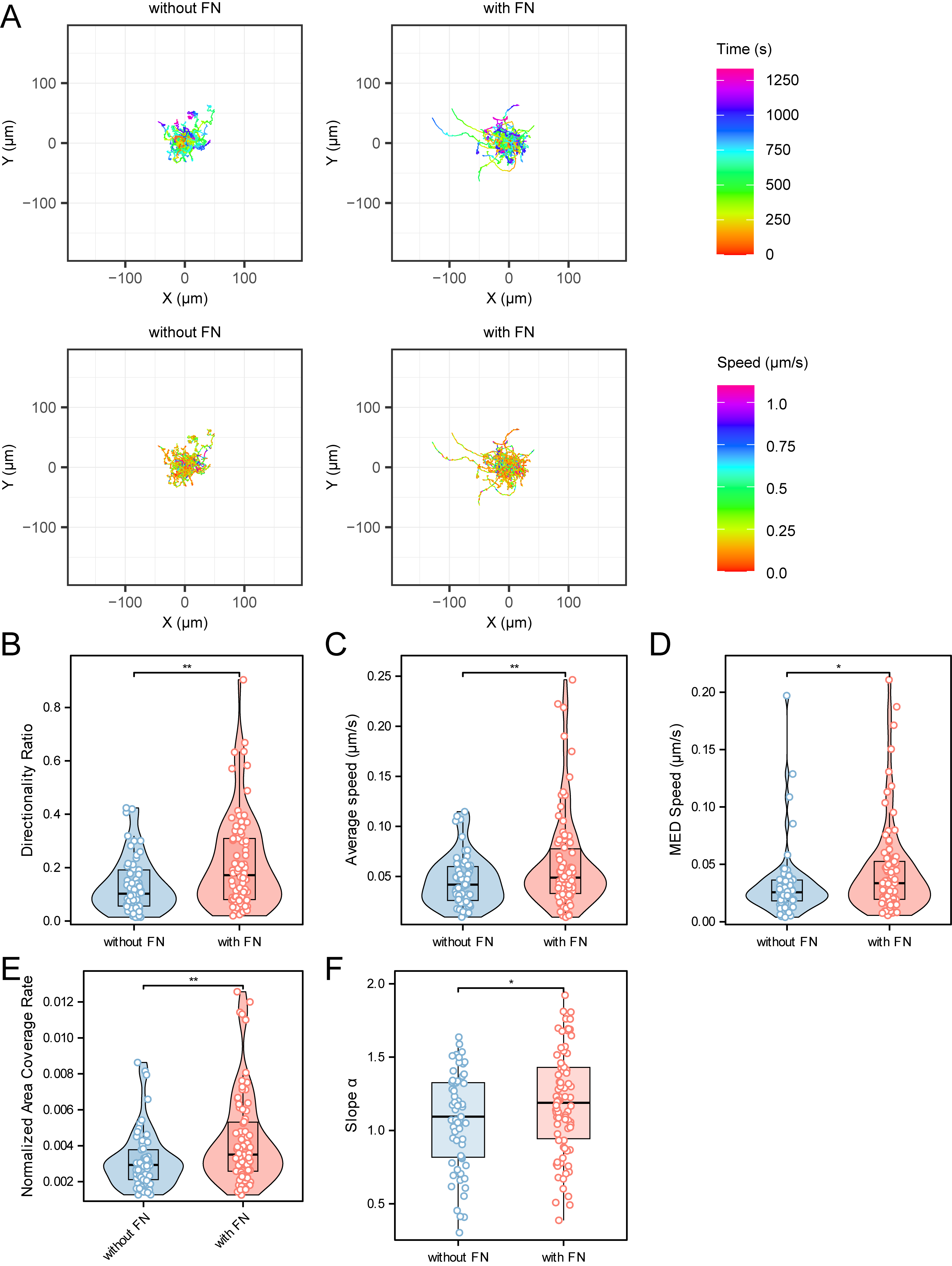

Supplement: S2 Fig — (A) Trajectory plots of trophozoites on fibronectin (FN)-coated (right panels, n = 82) and uncoated (left panels, n = 61) micropillars, with color mapping indicating time (top row) and the average speed over every four seconds between two consecutive frames (bottom row). (B-D) Quantitative comparisons of Directionality Ratio (B), Average Speed (C), MED speed (D), and Normalized Area Coverage Rate (by time and cell size) (E) of E. histolytica between migrating on fibronectin-coated and uncoated micropillars, with Mann-Whitney U tests. (F) Comparison of the slopes (α) of the fitted lines for the log(MSD)-log(time) plot (Fig 3G) representing individual cell migration under different conditions: fibronectin-coated (n = 82) versus uncoated micropillars (n = 61), with Mann-Whitney U test. * p < 0.05, ** P < 0.01. (TIF) [file ppat.1012618.s002.tif]

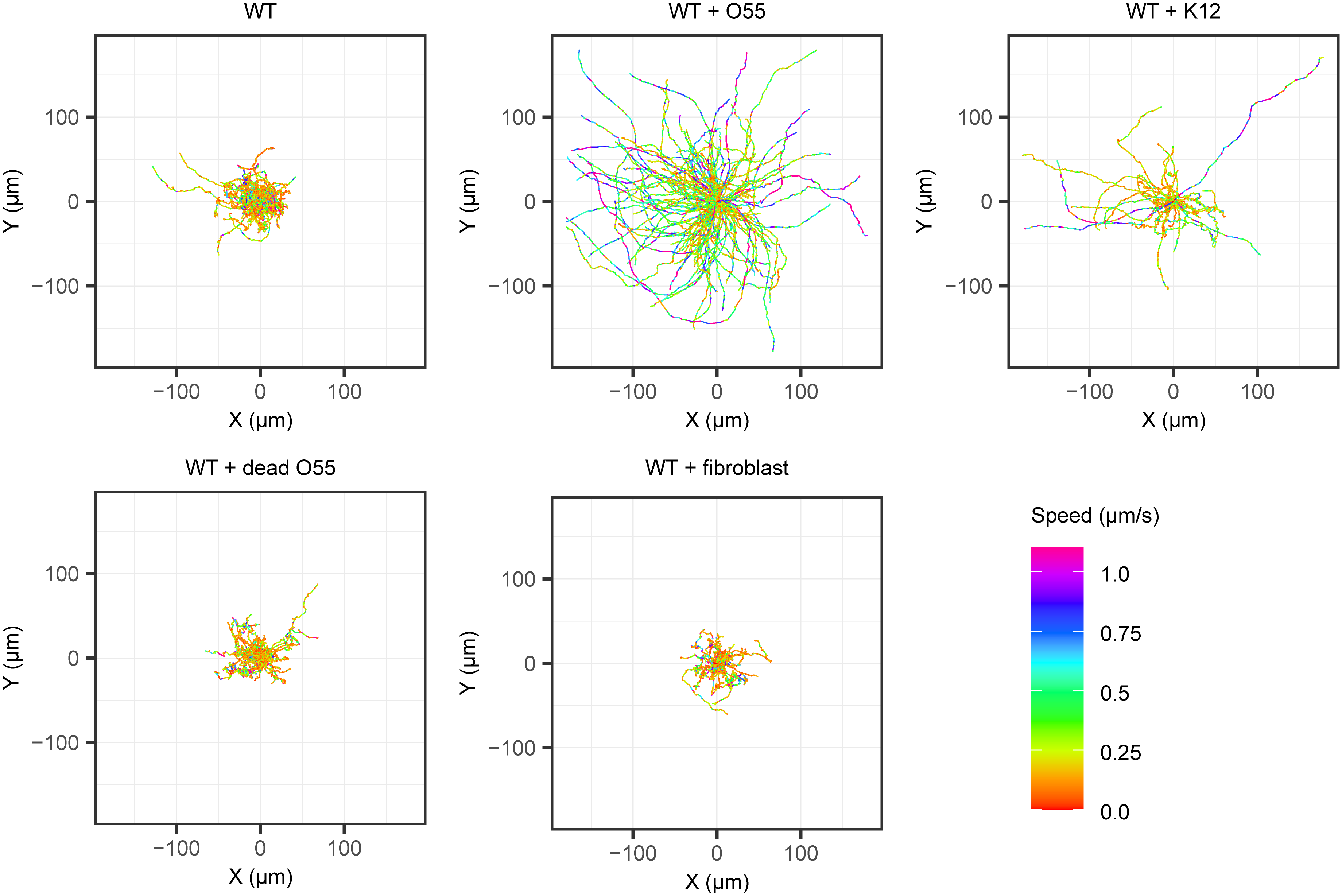

Supplement: S3 Fig — Noteworthily, O55 appears more frequently in cyan, blue, and purple (corresponding to higher speeds), while the other groups predominantly show red and yellow, clearly indicating that the O55 group moves faster. (TIF) [file ppat.1012618.s003.tif]

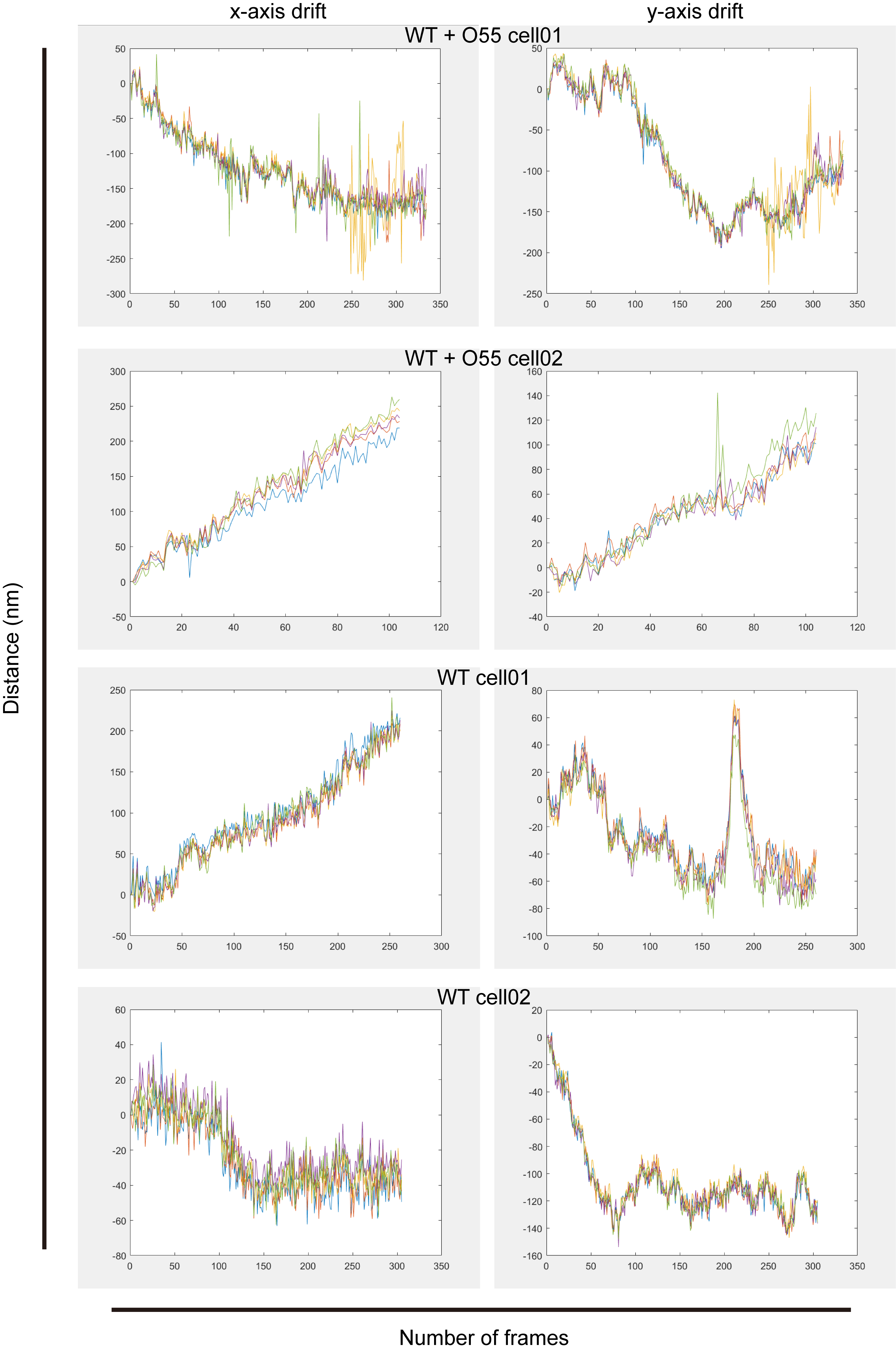

Supplement: S4 Fig — Five randomly selected micropillars, unaffected by any disturbances, were used for nano-tracking. Drift in both the x and y directions was calculated for each frame. Two examples of the drift for each the WT and WT + O55 groups in the x and y directions was illustrated. It can be observed that within a complete video of approximately 300 frames (4 seconds per frame), the drift is within 250 nm, which is less than 2 pixels, far smaller than the size of the cells and the distance the cells migrate within 300 frames. Note: resolution 0.144 μm/pixel, average cell size is 481.88 ± 6.65μm2 in area and 24.75 ± 0.17 μm in diameter. (TIF) [file ppat.1012618.s004.tif]

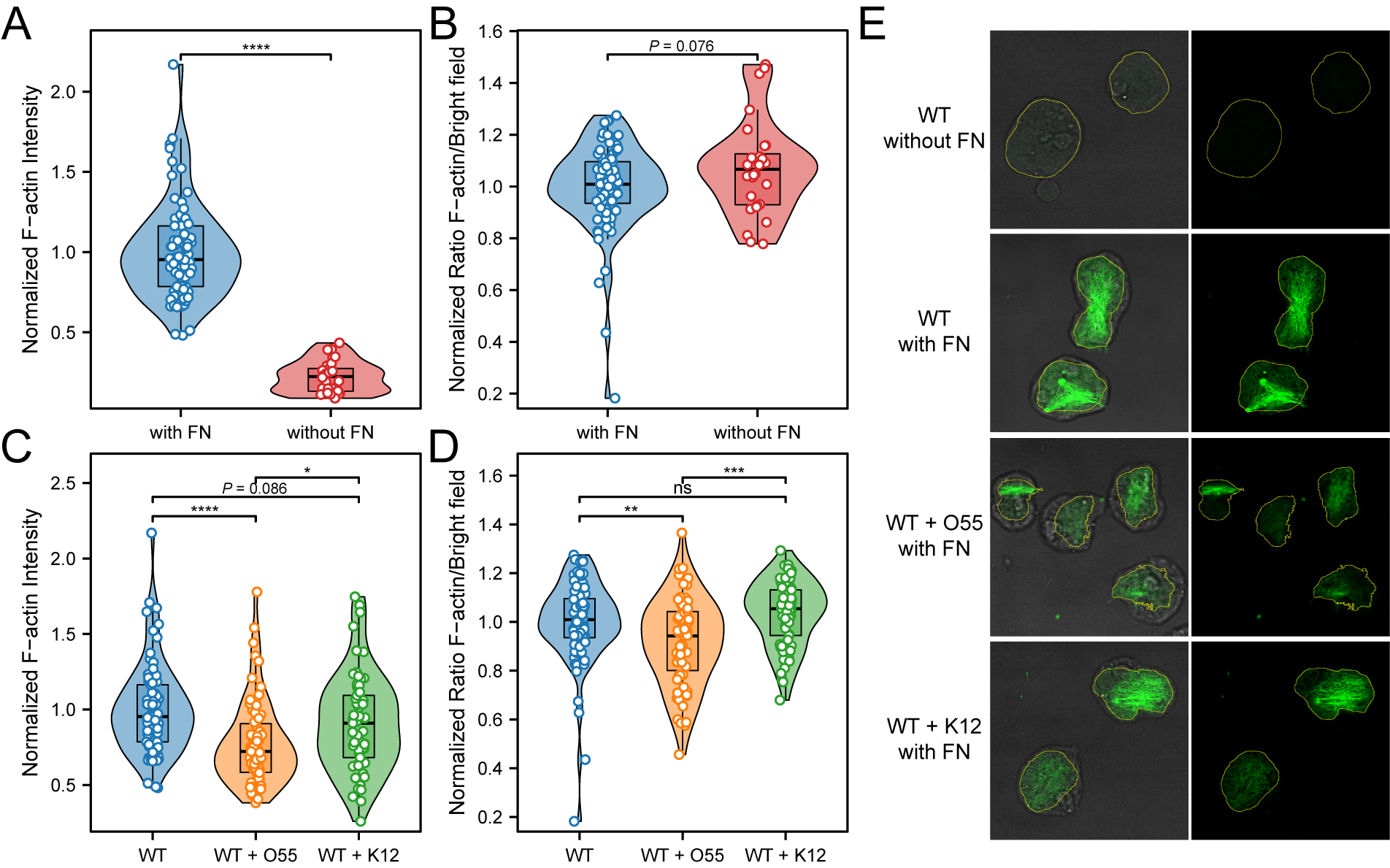

Supplement: S5 Fig — (A) Normalized Phalloidin intensity was quantitatively compared between E. histolytica trophozoites migrating on surface with (n = 77) and without (n = 28) fibronectin (FN) coating. (B) The normalized ratio of basal surface area (determined by confocal F-actin imaging) to the total area in the bright field (non-confocal imaging) was compared between trophozoites under two conditions of (A). (C) Phalloidin intensity was quantitatively compared among E. histolytica trophozoites under three conditions: control (n = 77), incubated with E. coli O55 (n = 67), and incubated with E. coli K12 (n = 61), on fibronectin (FN)-coated ibidi plates. (D) The normalized ratio of basal surface area (determined by confocal F-actin imaging) to the total area in the bright field (non-confocal imaging) was compared between trophozoites under three conditions of (C). (E) Representative images illustrate the differences in features compared in A-D. The yellow line outlines the basal surface area (determined by confocal F-actin imaging) of the cell. Statistical analyses, one-way ANOVA with post-hoc pairwise comparisons. * P < 0.05, ** P < 0.01, *** P < 0.001, **** P < 0.0001. (TIF) [file ppat.1012618.s005.tif]
